# The Social Intelligence Reliance Halo: Perceived Social Understanding, Epistemic Trust, and Reliance Quality in Human–AI Decision Making

**DOI:** 10.3390/jintelligence14100248

**Published:** 2026-10-08

**Authors:** Filiz Mizrak, Ayca Can Ozer Kirgiz, Hatice Gokce Demirel

**Affiliations:** 1 Management Information Systems, Atlas University, Istanbul 34408, Turkey; 2 Department of Business Administration, Istanbul Kent University, Istanbul 34433, Turkey; 3 Department of Business Administration, Fırat University, Elazig 23119, Turkey

**Keywords:** social intelligence reliance halo, human–AI decision making, epistemic trust, harmful overreliance, reliance quality

## Abstract

As generative AI systems become more socially responsive, personalized, and perspective-aware, a central question is whether such features improve human–AI decision quality or simply make AI advice more influential. This study introduces the Social Intelligence Reliance Halo as a proposed social-to-epistemic spillover pattern in which perceived social understanding functions as a non-diagnostic cue for epistemic evaluation: an AI that appears to understand the user may also be granted greater epistemic authority or reliability even when the relational cue provides no evidence of factual accuracy. A randomized two-condition experiment was conducted with 462 working adults with recent generative-AI experience. Participants interacted with either a High Social-Intelligence (High-SI) relational framing package or a Neutral AI across six repeated decision trials. Post-interaction measures assessed perceived social intelligence, epistemic authority, epistemic reliability, suspension of critical judgment, verification behavior, and AI literacy; trial-level behavioral reliance was analyzed with generalized estimating equations. The randomized High-SI package increased perceived social intelligence and increased advice-taking both when participants were initially correct and the contingent AI recommendation was wrong (OR = 1.507, *p* < .001) and when participants were initially incorrect and the recommendation was correct (OR = 1.800, *p* < .001). The Condition × participant-dependent advice-correctness interaction was nonsignificant (OR = 1.195, *p* = .289), providing no reliable evidence that the randomized treatment effect differed across these two states. Because recommendation correctness was mechanically determined by participants’ initial accuracy rather than independently randomized, this interaction is interpreted as exploratory treatment heterogeneity across participant-dependent correctness states rather than evidence that the High-SI package altered sensitivity to AI accuracy. Post-interaction perceived social intelligence was positively associated with epistemic authority and reliability, and AI literacy was associated with lower suspension of critical judgment and greater verification; these associations do not establish temporal or causal mediation. The study therefore provides experimental evidence that a bundled relational framing package can amplify both beneficial and harmful reliance, while the proposed social-to-epistemic pathway and accuracy-sensitive reliance remain questions for stronger future designs.

## 1. Introduction

Generative artificial intelligence is increasingly designed to appear socially responsive, personalized, and perspective-aware. Contemporary AI assistants can acknowledge users’ reasoning, adapt conversationally, and communicate in ways that resemble interpersonal understanding. Prior research shows that such human-like and anthropomorphic cues can influence trust, perceived humanness, compliance, and engagement with artificial agents (Araujo, 2018; de Visser et al., 2016; Waytz et al., 2014). Perceived social intelligence is particularly relevant because users may view an AI as capable of recognizing, anticipating, and adapting to human thoughts and behavior even without perceiving it as literally human (Barchard et al., 2020). Recent work further suggests that intelligence-related mental-state attributions can increase willingness to accept advice from large language models (Colombatto et al., 2025).

These developments are generally discussed as advantages for engagement, usability, and trust. However, they also raise a less examined problem: does feeling understood by AI lead users to make unwarranted inferences about what the AI knows? Perspective-sensitive communication provides no necessary evidence that an AI recommendation is factually accurate. Nevertheless, a user may infer that a system capable of understanding their reasoning is also more knowledgeable, dependable, or authoritative. Existing research establishes that social cues shape responses to AI and that beliefs about competence influence reliance, but these studies have largely developed separately. Social-cue and anthropomorphism research focuses on human-like interaction, trust research emphasizes acceptance and delegation, and algorithm-aversion and advice-taking research examines when users accept or reject automated recommendations (Dietvorst et al., 2015; Glikson & Woolley, 2020; Hoff & Bashir, 2015; Logg et al., 2019). The specific gap addressed here is therefore not whether social cues can increase trust, but whether perceived social understanding becomes an epistemic cue and whether the resulting behavioral influence is selectively warranted.

We address this gap by introducing the Social Intelligence Reliance Halo. We define the halo as a proposed cross-domain inferential pattern in which a relational impression—‘this AI understands my perspective’—becomes a cue for epistemic evaluation—‘this AI deserves weight’ or ‘this AI is likely to be accurate’—even when the relational cue contains no diagnostic information about factual correctness. The framework therefore links social/relational judgment to two more specific epistemic judgments: epistemic authority, or whether the AI’s conclusions deserve weight, and epistemic reliability, or whether its information is expected to be accurate and dependable. This formulation moves beyond generic trust by specifying both the triggering cue and the epistemic judgments to which that cue may spill over (Raviv et al., 1993; Jian et al., 2000). The Social Intelligence Reliance Halo is not treated as a single latent trait or standalone psychometric scale in the present study; it is a theoretical pattern examined through the conjunction of randomized framing effects, post-interaction construct associations, and behavioral reliance outcomes.

The behavioral consequences of this process require particular attention because greater reliance is not necessarily better reliance. Lee and See (2004) emphasized that the goal of automation should be appropriate reliance rather than maximal trust, while Parasuraman and Riley (1997) distinguished beneficial use from misuse and disuse. Similarly, research on algorithm appreciation and aversion shows that accepting or rejecting algorithmic advice is not inherently desirable; its value depends on whether the advice is actually useful (Dietvorst et al., 2015; Logg et al., 2019; Mahmud et al., 2022). Accordingly, the present study distinguishes appropriate reliance, in which users correct an initially wrong judgment by following accurate AI advice, from harmful overreliance, in which users abandon a correct judgment in favor of inaccurate AI advice. The contrast between these response rates distinguishes beneficial from harmful switching within the task. Because recommendation correctness is determined by participants’ initial accuracy rather than independently manipulated, however, this contrast should not be interpreted as showing whether users adjusted reliance in response to independently varying AI accuracy.

AI literacy may also be related to how users maintain epistemic vigilance in this process. Contemporary approaches conceptualize AI literacy as more than the ability to operate AI tools; it includes evaluating their capabilities, limitations, and appropriate uses (Laupichler et al., 2022; D. T. K. Ng et al., 2021; Wang et al., 2023). Greater AI literacy may be reflected in lower suspension of critical judgment and more frequent verification. Importantly, this does not necessarily imply immunity to socially intelligent cues. More AI-literate users may still perceive an AI as socially capable while remaining more willing to question or independently verify its recommendations.

Against this background, the present study asks whether a socially intelligent relational presentation increases users’ willingness to follow AI advice even when following that advice is harmful. We test this question in a randomized two-condition experiment with 462 working adults who had recent generative-AI experience. Participants completed six repeated population-comparison judgments while receiving either a High Social-Intelligence (High-SI) relational framing package or a Neutral AI message. The High-SI package acknowledged the participant’s perspective, affirmed the initial judgment as understandable, personalized the recommendation around that judgment, and employed a somewhat more elaborated relational style; the Neutral condition delivered the same substantive recommendation without this package. Because these features covaried, the randomized contrast identifies the effect of the package as a whole rather than the unique contribution of perspective taking, affirmation, personalization, warmth, or elaboration. On every trial, the AI recommended the unselected alternative. Recommendation correctness therefore depended mechanically on the participant’s initial accuracy: the recommendation was correct when the initial answer was wrong and incorrect when the initial answer was correct. This design permits direct observation of beneficial and harmful switching and an exploratory comparison of treatment effects across participant-dependent correctness states, but it does not independently manipulate AI accuracy and therefore cannot determine whether relational framing changes users’ responsiveness to recommendation accuracy.

The study’s novelty lies not in showing that social cues can increase trust or advice acceptance, which is already established, but in specifying a narrower social-to-epistemic spillover problem and connecting it to reliance quality. The study makes three contributions. First, it defines the Social Intelligence Reliance Halo as a proposed inferential pattern in which perceived social understanding can become a non-diagnostic cue for two distinct epistemic judgments: authority, or the weight granted to the AI as a source, and reliability, or the expected accuracy and dependability of its information. Second, it separates appropriate reliance from harmful overreliance, showing why greater advice acceptance cannot by itself be interpreted as better human–AI collaboration. Third, it distinguishes the evidence that the design can identify experimentally from the relationships it can examine only associationally: randomized assignment tests the behavioral influence of the High-SI package, whereas post-interaction psychological measures are used to assess whether the observed covariation is consistent with the proposed framework. The exploratory participant-dependent correctness comparison further motivates future work on accuracy-sensitive reliance, while leaving that question to studies in which AI accuracy is independently randomized. The resulting contribution is therefore a more precise account of when relational impressions may become epistemically consequential and why greater AI influence should not be equated with better reliance.

## 2. Literature Review and Hypothesis Development

### 2.1. Social Cognition, Anthropomorphism, and Human-like AI

People rarely interact with intelligent technologies as if they were completely neutral pieces of machinery. Once a system speaks fluently, responds contingently, remembers prior information, or appears to recognize what a person is trying to achieve, users begin to interpret its behavior through social categories that were originally developed for understanding other people. Anthropomorphism captures part of this tendency, but it is broader than simply giving a machine a human face or name. Epley et al. (2007) argued that anthropomorphism emerges when people apply human-centered knowledge to nonhuman agents, particularly when they seek understanding, predictability, or social connection. Mind-perception research similarly shows that people differ in the extent to which they attribute agency, experience, intention, and cognitive capacity to other entities (H. M. Gray et al., 2007; K. Gray & Wegner, 2012). In technological settings, these attributions matter because they shape users’ expectations about what an artificial agent can understand and accomplish. Waytz et al. (2014), for example, showed that anthropomorphizing an autonomous vehicle increased trust, while de Visser et al. (2016) found that anthropomorphic cognitive agents could exhibit greater resilience to trust violations. Together, these studies suggest that social interpretation is not an incidental response to AI; it can change how users evaluate and rely on the system.

This pattern is especially visible in conversational AI. Text-based agents can trigger social responses even without a physical embodiment because language itself communicates identity, responsiveness, and apparent intentionality. Araujo (2018) found that anthropomorphic design cues and the way conversational agency was framed influenced how users perceived both a chatbot and the organization behind it. Go and Sundar (2019) similarly showed that visual, identity, and conversational cues can increase perceived humanness, while Adam et al. (2021) demonstrated that chatbot design characteristics can influence user compliance in customer-service encounters. These effects are not universally beneficial. Crolic et al. (2022) showed that anthropomorphism can intensify negative reactions when a chatbot fails during a service interaction, indicating that humanization also raises expectations. Luo et al. (2019) further demonstrated that disclosure that an interaction partner was an AI chatbot could alter customer purchasing behavior. Taken together, this research suggests that people respond not only to what an AI says, but also to what kind of social actor they believe it to be. This distinction becomes increasingly important as generative AI systems are deliberately designed to sound attentive, personalized, emotionally aware, and socially responsive.

### 2.2. Perceived Social Intelligence and the Emergence of a Social-Intelligence Halo

Anthropomorphism alone does not fully capture the psychological response to contemporary generative AI. A system may appear socially capable even when users do not literally believe that it is human. What may matter more is whether the system seems able to recognize another person’s perspective, infer why a decision was made, anticipate reactions, and adapt its response accordingly. Barchard et al. (2020) conceptualized perceived social intelligence in terms of an artificial agent’s apparent ability to recognize, adapt to, and predict human behaviors, cognitions, and emotions, developing a broad family of measures for assessing these perceptions. More recent work reinforces the importance of these cues. Klein’s (2025) meta-analysis indicates that human-like social cues systematically affect social responses to text-based conversational agents, while Li and Sung (2021) link anthropomorphic AI interaction to reductions in psychological distance. These findings matter because users may interpret perspective-sensitive communication as evidence that the AI not only understands the person but also understands the underlying problem. Social intelligence may therefore become a basis for judgments that extend beyond the social domain. Recent LLM experiments also show that interface-level anthropomorphic and social cues can alter perceived anthropomorphism, perceived information accuracy or risk, and selected dimensions of trust (Cohn et al., 2024; Krämer et al., 2025).

Developments in large language models make this spillover especially plausible. Kosinski (2024) and Strachan et al. (2024) show that state-of-the-art language models can perform surprisingly well on tasks designed to assess aspects of theory of mind, although performance on such tasks should not be equated with human mental experience. More importantly for human–AI interaction, users themselves form judgments about whether an AI possesses capacities such as reasoning, planning, emotion, or understanding. Colombatto et al. (2025) provide particularly relevant evidence. In a preregistered experiment with 410 participants, they distinguished mental-state attributions related to intelligence from those related to experience and examined how these judgments related to actual acceptance of LLM advice. Intelligence-related attributions were strongly associated with advice acceptance, whereas experience-related attributions were not positively related to advice-taking. This result moves the literature beyond a simple “human-like AI produces more trust” explanation. It suggests that users discriminate among different perceived mental capacities and that competence-relevant attributions may be particularly important for reliance. The unresolved question is whether perceived social intelligence can generate a similar competence inference even when social understanding itself provides no diagnostic information about factual accuracy.

This possibility is conceptualized as a Social Intelligence Reliance Halo. The proposed halo occurs when cues signaling an understanding of the user’s perspective spill over into judgments about the AI’s epistemic competence. In shorthand, the perception that “this system understands why I think this way” may develop into the belief that “this system probably deserves greater epistemic weight” or “this system is probably more reliable.” The defining feature is therefore not social responsiveness alone, but a cross-domain inference from relational understanding to epistemic evaluation in the absence of corresponding diagnostic evidence about factual accuracy. Existing findings on social cues, perceived social intelligence, anthropomorphism, and mental-state attribution make such an inference theoretically plausible, but they do not establish whether it improves decision quality or simply increases receptivity to advice (Barchard et al., 2020; Colombatto et al., 2025; de Visser et al., 2016; Klein, 2025; Waytz et al., 2014). Accordingly, the framework treats perceived social intelligence as conceptually connected to more specific epistemic judgments rather than as an endpoint in itself. However, because the relevant psychological constructs were measured after the interaction, the observed associations do not establish temporal precedence among them.

The proposed halo is also conceptually distinct from several neighboring constructs. Anthropomorphism concerns attribution of human-like qualities to a nonhuman agent, whereas the halo does not require the AI to be perceived as human, conscious, or emotional (Araujo, 2018; Waytz et al., 2014). Generic trust concerns a broader willingness to accept vulnerability or depend on a system, whereas the halo specifies a relational cue and the particular epistemic judgments to which that cue may generalize (Hoff & Bashir, 2015; Jian et al., 2000). Algorithm appreciation and algorithm aversion describe greater or lesser receptivity to algorithmic advice, while advice-taking captures the behavioral use of a recommendation; neither concept by itself specifies why a cue about social understanding should alter perceived epistemic competence (Dietvorst et al., 2015; Logg et al., 2019). Automation bias describes inappropriate reliance and associated decision errors, whereas the halo is a proposed upstream inferential account that may contribute to such behavior without being equivalent to the behavior itself (Goddard et al., 2012; Parasuraman & Manzey, 2010). Finally, mental-state attribution concerns perceived capacities such as intelligence or experience; the halo focuses more narrowly on whether perceived social understanding spills into epistemic authority and reliability judgments (Colombatto et al., 2025). These distinctions position the Social Intelligence Reliance Halo as a specific social-to-epistemic spillover framework rather than a relabeling of established trust or reliance constructs.

### 2.3. From Social Understanding to Epistemic Authority and Reliability

Research on trust in automation provides an important foundation for this argument, but it also shows why a more precise conceptual vocabulary is needed. Trust has been examined as a major determinant of technology acceptance, cooperation, delegation, and reliance for decades (Hoff & Bashir, 2015; Madhavan & Wiegmann, 2007). Reviews of AI research similarly identify trust as a central mechanism shaping whether users accept recommendations and delegate decisions to intelligent systems (Bach et al., 2024; Glikson & Woolley, 2020; S. W. T. Ng & Zhang, 2025). Yet trust is not equivalent to reliance. Lee and See (2004) emphasized that the goal of automation design should be appropriate reliance, not simply greater trust, because excessive trust can produce misuse just as insufficient trust can produce disuse. Parasuraman and Riley (1997) made a related distinction among use, misuse, disuse, and abuse of automation. These frameworks are particularly relevant to generative AI because systems are increasingly able to produce responses that are socially persuasive even when their factual reliability varies. Increasing trust without improving the user’s ability to recognize those reliability boundaries may therefore create a new form of decision risk.

The distinction can be sharpened by asking what users believe the AI is trustworthy for. One possibility is epistemic authority: the system is treated as a source whose judgments deserve substantial weight. A second is epistemic reliability: the system is believed to provide accurate, dependable, or technically precise information. The epistemic-authority tradition conceptualizes such authority as the standing of a source in knowledge acquisition (Raviv et al., 1993), while trust-in-automation research identifies reliability and dependability as core components of trust in automated systems (Jian et al., 2000). The present framework therefore treats authority and reliability as related but distinct judgments and examines suspension of critical judgment separately as a vigilance outcome, consistent with broader work on epistemic vigilance (Sperber et al., 2010). Huynh and Aichner (2025) also highlight the importance of cognitive trust in generative AI, while Gulati et al. (2019) demonstrate that trust in human–computer interaction can be measured as a multidimensional psychological judgment. Shin (2021), meanwhile, shows that explainability and perceptions of causability shape trust and acceptance, underscoring that users form epistemic evaluations partly from the way a system presents and justifies its outputs.

The two constructs are therefore separated by the object of the judgment rather than by a claim that they are unrelated. Epistemic authority concerns source standing and deference: whether the AI’s judgment deserves weight, can function as evidence, or is treated as difficult to challenge. Epistemic reliability concerns an accuracy expectation: whether the AI is expected to provide factually correct, relevant, dependable, and technically precise information. A user may regard an AI as generally reliable yet still decline to grant it substantial decision authority or may defer to an apparently authoritative system while remaining uncertain about its accuracy in a particular case. Because both judgments concern epistemic evaluation, substantial empirical correlation is expected; the theoretical claim is that they are related dimensions with different meanings and potentially different links to vigilance and reliance, not orthogonal constructs.

A long tradition of automation research also demonstrates that these judgments evolve through experience. Dzindolet et al. (2002) showed that perceptions of the utility of automated aids influence whether users rely on them, while Dzindolet et al. (2003) directly linked trust to automation reliance. Lewandowsky et al. (2000) found that trust in automated systems develops dynamically rather than functioning as a fixed disposition. Importantly, users are also sensitive to the pattern and visibility of machine errors. Madhavan et al. (2006) showed that failures on tasks perceived as easy for humans can disproportionately undermine trust in automation. Conversely, anchoring on one’s own initial judgment can reduce subsequent reliance on automated diagnostic advice (Madhavan & Wiegmann, 2005). These findings demonstrate that reliance depends not only on objective system performance but also on how people interpret the system’s competence. The Social Intelligence Reliance Halo extends this logic by proposing that an apparently unrelated cue—social understanding—can become part of that competence judgment. If users interpret perspective-taking language as evidence of broader intelligence, they may grant the AI greater epistemic authority or reliability than the factual task itself warrants.

### 2.4. Epistemic Vigilance, Verification, and Suspension of Critical Judgment

Trustworthy decision making requires more than identifying a seemingly competent source. It also requires maintaining enough epistemic vigilance to evaluate when that source should be questioned. Advice-taking research has long shown that decision makers balance their own judgments against information supplied by others rather than simply replacing one judgment with another. Sniezek and Van Swol (2001) demonstrated that trust, confidence, and perceived expertise shape influence within judge–advisor systems, while Bonaccio and Dalal’s (2006) integrative review shows that advice use depends on characteristics of the decision maker, advisor, task, and interaction. Schrah et al. (2006) further connect advice use with information acquisition, and Rader et al. (2017) emphasize that advice operates as a form of social influence as well as a source of information. These insights are highly relevant to conversational AI because a recommendation presented through socially responsive language is simultaneously informational and interpersonal. Users may therefore respond not only to the content of the recommendation but to the perceived status of the source offering it.

Automation-bias research shows what can happen when this balance shifts too far. Goddard et al. (2012) document how automated decision support can contribute to commission and omission errors when users fail to independently evaluate automated outputs. Parasuraman and Manzey (2010) similarly describe complacency and automation bias as consequences of insufficient monitoring and attentional allocation, while Merritt et al. (2019) operationalize automation complacency in terms that include inadequate monitoring. In the generative-AI context, verification behavior represents a practical expression of resistance to such overreliance: users can cross-check factual claims, consult authoritative sources, inspect evidence, or seek independent confirmation. Earlier information-credibility research explicitly examined whether users verify online information and how verification behavior relates to credibility assessment (Flanagin & Metzger, 2000; Metzger et al., 2003). The present framework therefore distinguishes suspension of critical judgment, an internal tendency to accept or insufficiently challenge AI conclusions, from verification behavior, an active effort to test those conclusions. This distinction allows the study to examine whether epistemic trust is associated with greater receptivity, reduced scrutiny, reduced verification, or some combination of these processes. Recent behavioral evidence likewise shows that users can overrely on AI advice even when it conflicts with contextual information or their own assessment (Klingbeil et al., 2024).

### 2.5. From Advice Acceptance to Appropriate Reliance and Harmful Overreliance

The algorithm-advice literature presents a seemingly contradictory picture of human reliance. On one side, people sometimes avoid algorithms even when algorithmic forecasts are superior. Dietvorst et al. (2015) famously demonstrated algorithm aversion after people observed an algorithm make mistakes, and their later work showed that even a small degree of control over an imperfect algorithm can reduce that aversion (Dietvorst et al., 2018). Prahl and Van Swol (2017) likewise show that the discounting of automated advice depends on how the advisor and task are perceived. Castelo et al. (2019) demonstrate that algorithm aversion is strongly task dependent, while Burton et al. (2020) and Mahmud et al. (2022) synthesize a growing literature showing that aversion depends on factors such as task subjectivity, error experience, autonomy, expertise, and user expectations. Berger et al. (2021) further show that demonstrating an algorithm’s ability to learn can change these reactions, and Reich et al. (2023) identify learning from mistakes as another route for reducing algorithm aversion. Thus, resistance to AI advice is neither universal nor stable.

On the other side, users sometimes show algorithm appreciation, preferring algorithmic recommendations to human judgment. Logg et al. (2019) found that people can place substantial weight on algorithmic advice, particularly when they are not domain experts. This willingness to defer to AI also appears in consequential settings. Longoni et al. (2019) document resistance to medical AI but show that perceived uniqueness and personalization play important roles in that resistance. Lourenço et al. (2020) demonstrate that acceptance of financial robo-advice depends partly on perceptions of trust and expertise. Larkin et al. (2022) similarly examine willingness to accept AI advice for consequential risk decisions, while Kennedy et al. (2022) show that trust matters in public-policy algorithms. Schreuter et al. (2021) find that users may conform to conversational assistants, illustrating that conversational form can itself create social influence. Collectively, these studies suggest that the important question is no longer whether people trust algorithms more or less than humans. Rather, the key issue is when reliance is warranted. Recent generative-AI decision-support research similarly demonstrates that human-like system features and advice elaboration can alter trust and reliance, including inappropriate reliance on hallucinated advice (Spatscheck et al., 2024).

This shift becomes even more important in collaborative work. Jarrahi (2018) argues that human–AI decision making is best understood as a form of symbiosis in which human judgment and machine capabilities complement one another rather than one simply replacing the other. Sowa et al. (2021) reach a similar conclusion in managerial knowledge work, where AI systems increasingly operate as collaborative partners. Fügener et al. (2022), however, show that productive human–AI delegation faces cognitive challenges and that combining human and AI judgment does not automatically produce optimal outcomes. Chong et al. (2022) further demonstrate that confidence in oneself and confidence in AI evolve during interaction and influence whether AI advice is adopted. These findings point toward reliance quality as a more informative outcome than raw advice acceptance. If an AI is correct, following its recommendation may represent appropriate reliance. If the same user follows the AI when it is wrong despite having initially reached the correct answer, the identical behavioral act—switching to AI—becomes harmful overreliance. A measure that counts only total advice acceptance cannot distinguish these fundamentally different outcomes.

Accordingly, the present study separates behavioral reliance into two components. Appropriate reliance occurs when the user initially makes an incorrect judgment and then switches to correct AI advice. Harmful overreliance occurs when the user initially makes the correct judgment but abandons it after receiving incorrect AI advice. The contrast between these tendencies provides an exploratory within-design comparison of reliance quality across participant-dependent correctness states. Because recommendation correctness is determined by participants’ initial accuracy, it does not show whether users adjust reliance in response to independently varied AI accuracy. This approach also responds to a limitation in much of the trust and algorithm-aversion literature: increased reliance is often treated as evidence of successful adoption, while reduced reliance is treated as evidence of distrust. Yet neither is inherently desirable. Lee and See’s (2004) emphasis on appropriate reliance, Parasuraman and Riley’s (1997) distinction between use and misuse, and research on algorithm appreciation and aversion all point toward the same principle: a well-functioning human–AI relationship requires users to know when reliance is justified, not merely to rely more often (Dietvorst et al., 2015; Logg et al., 2019; Mahmud et al., 2022).

### 2.6. AI Literacy as an Epistemic Vigilance Resource

If socially intelligent AI can increase epistemic receptivity, users may require resources that help them preserve critical distance. AI literacy is increasingly conceptualized as more than knowing how to operate an AI tool. D. T. K. Ng et al. (2021) describe AI literacy as involving the ability to understand, use, evaluate, and ethically engage with artificial intelligence, while Laupichler et al. (2022) show that AI-literacy research in adult and higher education increasingly emphasizes critical evaluation alongside technical competence. Wang et al. (2023) operationalize this broader perspective in a validated AI Literacy Scale that includes users’ capacity to evaluate AI solutions and recognize appropriate uses and limitations. Such competencies should be particularly important in generative-AI environments, where outputs may sound fluent and confident even when they are incorrect. Users who understand that linguistic sophistication is not equivalent to factual reliability may be better positioned to resist unjustified epistemic inferences.

This reasoning suggests that AI literacy may function as an epistemic-vigilance resource, although the present study evaluates this possibility through associations rather than through a causal intervention on literacy. A literate user may still perceive an AI as socially intelligent and may even acknowledge its usefulness, while remaining more willing to question, cross-check, or independently verify its advice. In this sense, AI literacy can function as an epistemic-vigilance resource rather than as a generalized form of skepticism. This distinction is important because indiscriminate distrust would merely reproduce algorithm aversion, whereas critical AI literacy should support selective reliance. The present framework therefore expects greater AI literacy to be associated with lower suspension of critical judgment and greater verification behavior rather than assuming that knowledgeable users are simply immune to the persuasive effects of socially intelligent AI. This interpretation connects the educational AI-literacy literature with the broader trust and automation literature and provides a direct managerial implication: organizations may need to develop employees’ ability to evaluate AI, not simply their ability to use it.

### 2.7. Integrating the Literature: The Social Intelligence Reliance Halo

Taken together, the literature identifies most of the pieces of the problem, but they have largely been studied separately. Anthropomorphism research shows that human-like cues alter trust and social responses (Araujo, 2018; de Visser et al., 2016; Waytz et al., 2014). Social-intelligence and theory-of-mind research shows that users and researchers attribute differentiated social-cognitive capacities to artificial agents (Barchard et al., 2020; Kosinski, 2024; Strachan et al., 2024). Trust research demonstrates that beliefs about system competence are related to reliance (Glikson & Woolley, 2020; Hoff & Bashir, 2015), while automation-bias and advice-taking research shows that reliance can become problematic when independent monitoring is reduced (Bonaccio & Dalal, 2006; Goddard et al., 2012; Parasuraman & Manzey, 2010). Algorithm-aversion and algorithm-appreciation research further demonstrates that users can both under-rely and over-rely on artificial advice depending on task and context (Dietvorst et al., 2015; Logg et al., 2019). What remains less developed is an integrated account of a specific inferential error: a socially favorable impression being carried across domains and used as evidence of epistemic competence even when it is not diagnostic of factual accuracy.

The Social Intelligence Reliance Halo addresses that gap by specifying three conceptually distinct elements: the relational cue, the epistemic inference, and the reliance consequence. The relational element concerns perceived social understanding; the epistemic element concerns authority and reliability judgments; and the behavioral element concerns how much weight is ultimately given to AI advice. The halo is the proposed spillover connecting these domains, not any one of the component constructs. This distinction is important because a person can anthropomorphize an AI without granting it epistemic authority, can trust an AI for one purpose without perceiving social understanding, can appreciate algorithmic advice without relying on social cues, and can display automation bias for reasons unrelated to social intelligence. By separating these constructs, the framework identifies the particular inferential bridge that is theoretically novel: relational understanding may be treated as evidence about epistemic competence despite lacking diagnostic value for the factual task.

This formulation also clarifies the evidentiary status of the present study. The Social Intelligence Reliance Halo is proposed as a theoretical pattern rather than estimated as a single latent factor. The randomized condition provides evidence about the behavioral influence of the High-SI relational framing package; the post-interaction measures examine whether perceived social intelligence covaries with epistemic authority, reliability, and vigilance-related constructs; and the behavioral task separates beneficial from harmful reliance opportunities. Convergence across these components can be consistent with the halo framework, but the present design does not establish a temporally ordered psychological mechanism. This narrower claim preserves the framework’s novelty without treating adjacent constructs or associational pathways as if they were equivalent to the halo itself.

Most importantly, the halo framework raises a behavioral question that differs from the usual adoption logic and from simple algorithm appreciation. Greater willingness to follow AI is not inherently desirable; the normative issue is whether influence tracks when advice deserves to be followed. In principle, socially intelligent presentation could produce more accuracy-sensitive reliance if it increased reliance more strongly when AI advice is correct than when it is wrong, or it could function as a generalized reliance cue that increases receptivity across both situations. Colombatto et al. (2025) already show that intelligence-related mental-state attributions are associated with greater LLM advice acceptance, making it insufficient to argue merely that perceived intelligence or social capability increases trust. The more consequential question is whether the social-to-epistemic spillover changes reliance selectively or indiscriminately. In the present design, recommendation correctness is participant-dependent rather than independently randomized. The study can therefore distinguish beneficial from harmful reliance and examine treatment-effect heterogeneity across those states, but it cannot determine whether the relational framing changes responsiveness to independently varied AI accuracy. It tests whether a relationally framed AI becomes more behaviorally influential, including in situations where following its contingent recommendation is harmful.

**Figure 1:**
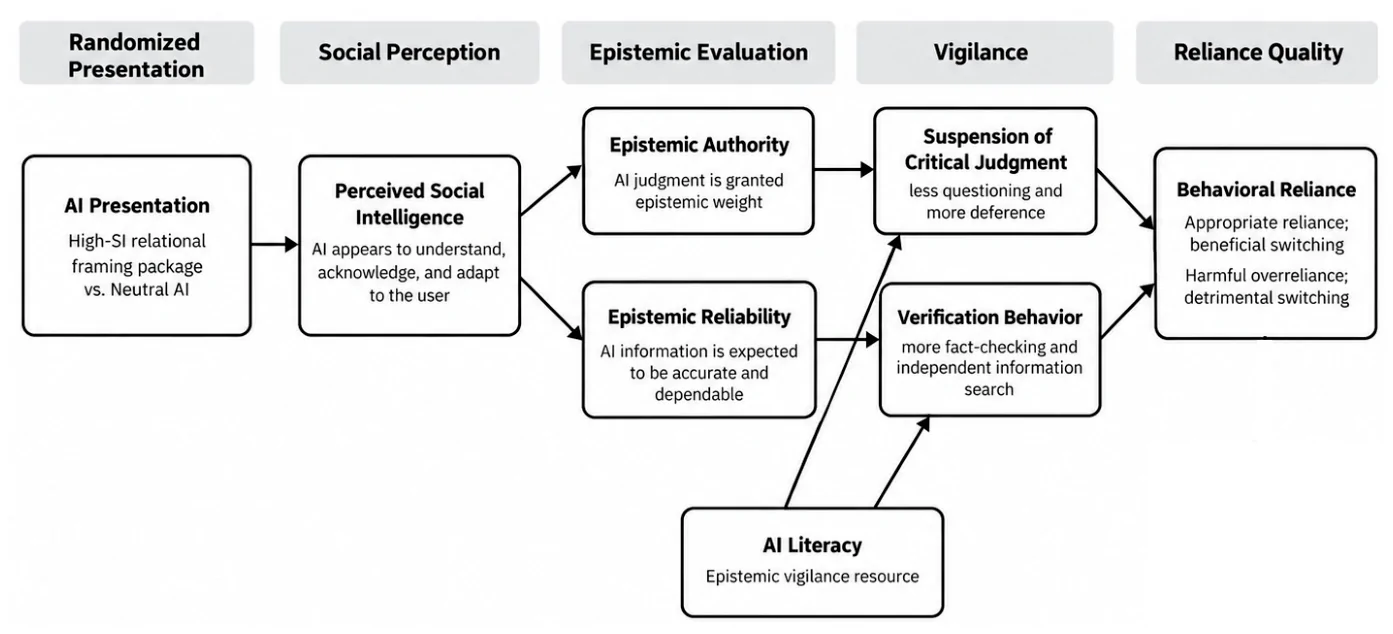
Conceptual Model of the Social Intelligence Reliance Halo. The High-SI node denotes the bundled relational framing package. Except for the randomized package-to-perceived-social-intelligence path, arrows represent theoretical associations rather than established temporal or causal pathways. Reliance quality is represented through appropriate reliance and harmful overreliance; responsiveness to independently varied AI accuracy is not modeled as an empirically tested outcome.

Figure 1 integrates these arguments into the proposed Social Intelligence Reliance Halo framework. The arrows represent theoretical propositions linking the randomized High-SI relational framing package, perceived social intelligence, epistemic judgments, vigilance-related constructs, and behavioral reliance; except for experimentally randomized condition effects, they should not be read as established temporal or causal paths. Reliance quality is represented through the distinction between appropriate reliance and harmful overreliance, without implying that responsiveness to independently manipulated AI accuracy was tested.

### 2.8. Confirmatory Hypotheses and Exploratory Research Questions

Building on the preceding literature, the proposed framework distinguishes three linked stages: social/relational perception, epistemic evaluation, and behavioral reliance. Research on anthropomorphism and socially responsive artificial agents shows that human-like and perspective-sensitive cues can influence how users perceive, trust, and respond to artificial systems (Araujo, 2018; Barchard et al., 2020; de Visser et al., 2016; Waytz et al., 2014). More recent evidence further indicates that competence-related mental-state attributions can increase acceptance of advice from large language models (Colombatto et al., 2025). However, trust and reliance are not equivalent, and greater acceptance of automated advice does not necessarily indicate better decision making (Lee & See, 2004; Parasuraman & Riley, 1997). Accordingly, the analyses are divided into confirmatory hypotheses and exploratory research questions. H1, H5, and H6 concern effects of the randomized High-SI relational framing package. H2a–H4 are theory-specified confirmatory association hypotheses among post-interaction psychological constructs; because those constructs were measured after the behavioral trials, these hypotheses test covariation only and do not establish temporal or causal pathways. ERQ1 and ERQ2 address questions for which the present design does not support confirmatory causal inference: treatment heterogeneity across participant-dependent advice-correctness states and compound post-interaction associations with harmful overreliance, respectively.

Confirmatory hypotheses

**H1.**  *The High Social-Intelligence relational framing package increases perceived social intelligence. This expectation follows from research showing that users infer social capabilities from conversational, anthropomorphic, perspective-sensitive, and human-like interaction cues *(Araujo, 2018; Barchard et al., 2020; Cohn et al., 2024; Klein, 2025; Krämer et al., 2025)*. *Barchard et al. (2020)*, in particular, conceptualize perceived social intelligence in terms of an artificial agent’s apparent capacity to recognize, predict, and adapt to human thoughts and behavior.*

**H2a.**  *Perceived social intelligence is positively associated with epistemic authority. Socially favorable perceptions may extend beyond relational evaluation and covary with the weight assigned to an AI’s judgments. The epistemic-authority literature conceptualizes authority as the standing granted to a source in processes of knowledge acquisition, while AI and automation research shows that perceptions of competence can influence reliance on technological advice *(Raviv et al., 1993; Glikson & Woolley, 2020; Hoff & Bashir, 2015)*. *Colombatto et al. (2025)* further show that intelligence-related mental-state attributions are associated with greater acceptance of LLM advice.*

**H2b.**  *Perceived social intelligence is positively associated with perceived epistemic reliability. Reliability and dependability are established dimensions of trust in automated systems *(Jian et al., 2000)*. If users interpret an AI’s apparent understanding of their perspective as evidence of broader intelligence, social judgments may spill over into assessments of whether the system’s information is accurate and dependable *(Jian et al., 2000; Glikson & Woolley, 2020; Colombatto et al., 2025)*. This proposed spillover constitutes a central element of the Social Intelligence Reliance Halo.*

**H3a.**  *Epistemic authority is positively associated with suspension of critical judgment. Advice-taking research suggests that perceived expertise and source status affect the weight assigned to external recommendations *(Bonaccio & Dalal, 2006; Sniezek & Van Swol, 2001)*. When a source is granted substantial epistemic authority, users may be less inclined to challenge its conclusions independently. This reasoning is consistent with research on epistemic vigilance and automation bias, which emphasizes the importance of maintaining scrutiny when evaluating information supplied by apparently competent sources *(Goddard et al., 2012; Parasuraman & Manzey, 2010; Sperber et al., 2010)*.*

**H3b.**  *Perceived epistemic reliability is positively associated with suspension of critical judgment. Users who regard an AI system as accurate, dependable, and technically reliable may perceive less need to independently question its recommendations. Prior automation research shows that trust and perceived utility affect reliance, while excessive confidence in automated systems may contribute to reduced monitoring and complacency *(Dzindolet et al., 2002, 2003; Merritt et al., 2019; Parasuraman & Manzey, 2010)*.*

**H4.**  *Greater AI literacy is associated with stronger epistemic vigilance, reflected in lower suspension of critical judgment and greater verification behavior. AI literacy increasingly encompasses the ability not merely to operate AI systems but also to evaluate their capabilities, limitations, appropriate applications, and outputs critically *(Laupichler et al., 2022; D. T. K. Ng et al., 2021; Wang et al., 2023)*. Such evaluative competence should be reflected in greater scrutiny and more frequent independent verification rather than uncritical acceptance of fluent or confident AI output.*

**H5.**  *The High Social-Intelligence relational framing package increases harmful overreliance on trials in which participants are initially correct and the AI’s contingent recommendation is therefore incorrect. Human-like and socially responsive cues can increase trust, compliance, and conformity toward artificial systems *(Adam et al., 2021; de Visser et al., 2016; Schreuter et al., 2021; Waytz et al., 2014)*. When such heightened receptivity is not matched by greater attention to factual accuracy, users may follow erroneous automated recommendations, consistent with established concerns regarding automation bias and misuse *(Goddard et al., 2012; Klingbeil et al., 2024; Parasuraman & Manzey, 2010; Parasuraman & Riley, 1997; Spatscheck et al., 2024)*.*

**H6.**  *The High Social-Intelligence relational framing package increases appropriate reliance on trials in which participants are initially incorrect and the contingent AI recommendation is therefore correct. The same social cues that make an AI more influential may also increase acceptance when its advice is beneficial. Prior research demonstrates algorithm appreciation, substantial weighting of algorithmic advice, and greater acceptance when systems are perceived as trustworthy or competent *(Logg et al., 2019; Lourenço et al., 2020; Schreuter et al., 2021)*. Accordingly, the High-SI relational framing package is expected to increase willingness to revise an incorrect initial judgment in favor of correct AI advice.*

Exploratory research questions

The following analyses are explicitly exploratory and are not treated as confirmatory tests of responsiveness to independently varied AI accuracy, causal mediation, or the full psychological mechanism proposed by the framework.

- Exploratory Research Question 1 (ERQ1): Does the effect of the High Social-Intelligence relational framing package on behavioral reliance differ between initially correct trials, on which the contingent AI recommendation is incorrect, and initially incorrect trials, on which the contingent AI recommendation is correct? The critical issue is not simply whether users follow AI advice more often, but whether the randomized treatment effect varies across participant-dependent correctness states in a manner relevant to the quality of reliance. Research on appropriate reliance argues that maximizing trust is not itself desirable; effective human–automation interaction requires users to rely when reliance is warranted and resist when it is not (Lee & See, 2004; Parasuraman & Riley, 1997). Research on human–AI collaboration similarly indicates that greater reliance does not automatically translate into superior decision performance (Chong et al., 2022; Fügener et al., 2022). However, participant-dependent recommendation correctness was not independently manipulated or randomized. Because the AI always recommended the unselected alternative, correctness followed mechanically from participants’ initial accuracy. The interaction therefore evaluates treatment-effect heterogeneity across participant-dependent correctness states rather than whether users adjust reliance to experimentally varied AI accuracy. A direct test of accuracy-sensitive reliance would require independent manipulation of recommendation correctness. A recent LLM experiment provides a useful design contrast: McGrath et al. (2024) systematically varied whether AI-assistant recommendations were accurate or inaccurate, allowing accuracy-related changes in trust and reliance to be examined independently of participants’ initial answers.
- Exploratory Research Question 2 (ERQ2): Do post-interaction perceived social intelligence, epistemic authority, epistemic reliability, and vigilance variables display compound statistical associations with harmful overreliance that are consistent with the proposed Social Intelligence Reliance Halo framework? The theoretical basis for this exploratory question combines evidence that social and competence-related impressions are related to reliance (Barchard et al., 2020; Colombatto et al., 2025), that epistemic authority and perceived reliability shape how information sources are evaluated (Jian et al., 2000; Raviv et al., 1993), and that reduced monitoring and verification are associated with inappropriate automation reliance (Goddard et al., 2012; Merritt et al., 2019; Parasuraman & Manzey, 2010; Sperber et al., 2010). Because all focal psychological variables were measured after the six behavioral trials, ERQ2 concerns post-interaction covariation only and does not test temporal precedence or causal mediation.

## 3. Method

### 3.1. Research Design

The study used a randomized two-condition between-subjects experiment to estimate the effect of a High Social-Intelligence relational framing package relative to a Neutral AI message on perceived social intelligence and behavioral reliance, while examining the remaining post-interaction epistemic and vigilance measures associationally. Participants were assigned to either the High-SI or Neutral condition. The High-SI package simultaneously acknowledged the participant’s perspective, affirmed the initial judgment as understandable, personalized the recommendation around that judgment, and used a warmer, somewhat more elaborated relational style. The Neutral response recorded the choice and provided the same substantive recommendation without this package of relational cues. The recommendation target, factual criterion, task, and substantive decision information were held constant. However, the relational features, evaluative tone, and degree of elaboration were not experimentally separated from one another. Randomization therefore identifies the average effect of the complete High-SI relational framing package relative to the Neutral comparator; it does not identify the unique causal effect of perceived social understanding, perspective taking, affirmation, personalization, warmth, positive feedback, message length, or elaboration individually.

Participants completed six repeated population-judgment trials. On each trial they made an initial choice, rated confidence from 0 to 100, received an AI recommendation for the unselected alternative, made a final choice, and rated confidence again. Because the AI always recommended the unselected option, its advice was objectively correct when the participant’s initial answer was wrong and objectively incorrect when the initial answer was correct. This design made it possible to distinguish appropriate reliance from harmful overreliance and to compare whether the randomized High-SI package effect differed between the two participant-dependent correctness states. Because those states were determined by participants’ initial accuracy rather than by an independent manipulation of AI accuracy, the comparison was treated as exploratory and not as a test of responsiveness to independently varied AI accuracy.

### 3.2. Participants and Screening

Data collection took place from April through May 2026. The main study targeted working adults with recent generative-AI experience. Participants were recruited through the researchers’ professional LinkedIn network and through email invitations distributed to employees at relevant companies. Participation was voluntary and no monetary or other compensation was provided. The study protocol was reviewed and approved by the university ethics committee prior to data collection. Before proceeding, participants viewed the study information and provided electronic informed consent by selecting “I agree” to the statement: “By selecting I agree, you confirm that you are at least 18 years old, have read the study information, and voluntarily agree to participate.” Eligibility required age of at least 18 years, current employment, and use of a generative AI system during the previous three months. The finalized survey was distributed to 650 prospective participants, of whom 482 completed the study (74.2%).

Responses were screened for eligibility, duplicate identifiers and response patterns, missing required data, out-of-range values, behavioral-task missingness, straight-lining, and the instructed-response attention check. Twenty respondents (4.1% of completed responses) failed the attention check and were excluded; no exclusions were required for the other screening criteria. The final analytic sample was *N* = 462 (95.9% of completed responses; 71.1% of invitations).

The final condition allocation remained balanced, with 230 participants in the Neutral condition (49.8%) and 232 in the High-SI condition (50.2%). Participants ranged from 21 to 68 years (*M* = 39.76, *SD* = 10.85); 53.2% were men, 43.1% women, 1.7% nonbinary, and 1.9% preferred not to report gender. Most held at least a bachelor’s degree, and 52.2% reported managerial responsibility. The sample represented diverse professional fields and countries of residence, with 58.2% residing in the United States. All retained participants had recent GenAI experience: 32.3% used GenAI several times per week, 25.8% about once per day, and 20.8% several times per day. ChatGPT was the most frequently reported primary tool (56.9%).

### 3.3. Experimental Decision Tasks

Behavioral reliance was assessed with six paired-country population judgments summarized in Table 1, Panel A. The comparisons were selected to yield nontrivial judgments, and pilot testing confirmed moderate initial difficulty across all six tasks. In each trial, participants first selected the country they believed had the larger 2025 population and rated their confidence. They then viewed the condition-specific AI message recommending the alternative country, made a final decision, and provided a second confidence rating.

The recommendation rule was intentionally contingent on the participant’s initial choice rather than on a fixed AI accuracy schedule. Thus, when the initial response was incorrect, switching to the recommended alternative corrected the decision; when the initial response was correct, switching produced an error. This within-task logic generated repeated opportunities to observe beneficial and harmful reliance under the same experimental framing. However, it also means that advice correctness was participant-dependent: correct and incorrect recommendations arose from participants’ initial accuracy, not an independent AI-accuracy assignment. Accordingly, comparisons across these states describe treatment-effect heterogeneity within the task design and cannot establish whether reliance responds to independently varied AI accuracy. In the 30-person pilot, initial accuracy across the six tasks ranged from 56.7% to 76.7% and switching rates from 33.3% to 50.0%; no task was excessively easy or difficult, so all six were retained.

### 3.4. Experimental Manipulation

The manipulation scripts are reproduced in Table 1, Panel B. In the High-SI relational framing package, the AI validated the participant’s initial judgment, acknowledged the participant’s perspective, tailored the wording around the participant’s initial choice, and then recommended the alternative. In the Neutral condition, the AI recorded the initial choice, restated the population criterion, and recommended the alternative. The recommendation target, factual criterion, and substantive task information were identical across conditions. Nevertheless, the High-SI message differed from the Neutral message on several features at once: perspective acknowledgment, affirmation/positive feedback, personalization, relational warmth or tone, and degree of elaboration; it was also somewhat longer. These features were intentionally bundled rather than factorially isolated. Accordingly, throughout the manuscript the treatment is interpreted as a High-SI relational framing package. Any observed condition effect may reflect one component, several components, their interaction, or the greater elaboration of the message, and cannot be attributed uniquely to ‘social intelligence’ or social understanding per se.

The manipulation package was examined before the main study in the separate 30-person pilot. Perceived social intelligence was higher in the High-SI condition (*M* = 3.88) than in the Neutral condition (*M* = 3.23), *t* = 2.23, *p* = .034, *d* = 0.81, supporting use of the finalized scripts. This pilot establishes that the package produced the intended difference in perceived social intelligence at the condition level; it does not identify which bundled component generated that perception. The confirmatory manipulation check in the main analytic sample is reported in Section 4.2.

### 3.5. Measures

The questionnaire measured six focal constructs: evaluative AI literacy (AIL), perceived social intelligence (PSI), epistemic authority attribution (EA), perceived epistemic reliability (ER), suspension of critical judgment (SCJ), and verification behavior (VB). Before fielding, three experts with backgrounds in social cognition/human–AI interaction, management/organizational behavior, and quantitative methods/psychometrics reviewed the measures and stimuli for construct relevance, clarity, response-scale appropriateness, and realism. The EA items were study-developed for the generative-AI context with reference to the epistemic-authority tradition (Raviv et al., 1993) and operationalized source standing, decisional weight, evidentiary status, and willingness to challenge the source. ER items were informed by established trust-in-automation work on reliability and dependability (Jian et al., 2000) and operationalized expected factual accuracy, relevance, dependability, and accuracy inferred from technical precision. This content distinction was intentional: EA asks how much epistemic weight the source is granted, whereas ER asks how accurate and dependable its information is expected to be. SCJ items drew on epistemic-vigilance and automation-complacency research (Sperber et al., 2010; Merritt et al., 2019), and VB items were informed by prior information-verification research (Flanagin & Metzger, 2000; Metzger et al., 2003). The scale-level content-validity index was 0.963, and four items or stimuli were modified for wording clarity. A separate 30-person pilot produced Cronbach’s alpha values from 0.704 to 0.884 across the six focal scales.

Table 1, Panel C reports the full wording, response format, scoring, and source of the 27 indicators used in the measurement model. AIL comprised three 1–7 items; PSI comprised eight 1–5 items, with PSI8 reverse-coded before scoring; EA, ER, and SCJ each comprised four 1–6 items; and VB comprised four 1–5 frequency items. For each construct, the composite score was the arithmetic mean of its constituent items so that scores remained on the original response metric. Higher AIL and VB scores indicated stronger evaluative literacy and verification, whereas higher SCJ indicated greater suspension of independent scrutiny. All focal psychological measures were administered after participants completed the six behavioral decision trials. Accordingly, their relationships with earlier behavioral reliance are interpreted as post-interaction associations rather than as temporally ordered mediators. Panel D reports the supplementary post-task reaction/manipulation-check items and the attention check. Demographic and background variables included age, gender, education, professional experience, managerial status, professional field, country of residence, decision responsibility, primary GenAI tool, and general, work-related, and decision-related GenAI-use frequency.

Table 1 consolidates the experimental decision tasks, condition-specific manipulation scripts, focal survey measures, and supplementary reaction and quality-control items used in the study. Complete questionnaire items, response scales, coding rules, and scoring details are provided in Table S1 of the Supporting Information.

### 3.6. Behavioral Outcomes

Behavioral reliance was derived from the initial and final responses on the six decision trials. A switch was recorded when the participant changed from the initially selected country to the AI-recommended alternative. Appropriate reliance was defined on initially incorrect trials, where switching moved the participant to the correct answer; the respondent-level Appropriate Reliance Rate was the number of such switches divided by the number of initially incorrect trials. Harmful overreliance was defined on initially correct trials, where switching moved the participant to an incorrect answer; the Harmful Overreliance Rate was the number of harmful switches divided by the number of initially correct trials.

A respondent contributed to a rate only when at least one trial was eligible for that outcome. Trial-level analyses therefore used the eligible decisions directly rather than requiring both respondent-level rates to be estimable. For an exploratory respondent-level summary of reliance quality, an index was computed as Appropriate Reliance Rate minus Harmful Overreliance Rate. Positive values indicate relatively greater switching when the contingent recommendation is beneficial than when it is harmful within this participant-dependent design, whereas values near zero indicate similar receptivity across the two states. Because recommendation correctness was mechanically determined by participants’ initial accuracy, this index is not treated as a measure of responsiveness to independently varied AI accuracy. The distinction remains useful for separating increases in beneficial reliance from increases in harmful overreliance.

### 3.7. Analytical Strategy

The analytical sequence was designed to separate confirmatory and exploratory questions that require different forms of evidence. Confirmatory analyses comprised measurement validation; the randomized condition effect on perceived social intelligence (H1); theory-specified post-interaction association hypotheses among PSI, EA, ER, SCJ, VB, and AI literacy (H2a–H4); and the randomized High-SI package effects on harmful and appropriate reliance opportunities (H5–H6). Exploratory analyses comprised the bootstrap compound-association analysis addressing ERQ2, the Condition × participant-dependent correctness interaction addressing ERQ1, the respondent-level reliance-quality contrast, and robustness checks. Analyses therefore proceeded from measurement validation to standardized construct-association models, exploratory bootstrap compound-association analysis, trial-level generalized estimating equations (GEE), the exploratory treatment-heterogeneity interaction, the exploratory respondent-level reliance-quality contrast, and robustness checks. Throughout the manuscript, coefficients involving randomized condition assignment are described as experimental treatment effects, whereas relationships among measured psychological constructs are described as associations; theoretical arrows are treated as propositions rather than established causal pathways. Because PSI, EA, ER, SCJ, and VB were measured after completion of the six decision trials, their relations with earlier behavioral outcomes cannot establish temporal precedence or causal mediation. The final screened sample (*N* = 462) was used for the measurement and construct-level analyses; behavioral models used all eligible decision trials contributed by those respondents.

Measurement quality was evaluated before testing the theory-specified construct-association hypotheses because path estimates are interpretable only if the constructs display adequate internal consistency and convergent validity. Internal consistency was assessed using Cronbach’s *α,* corrected item-total correlations, and composite reliability (CR), while average variance extracted (AVE) summarized the proportion of indicator variance captured by each construct. The principal reliability quantities were defined as follows, where *k* denotes the number of items, $\sigma_{i}^{2}$ the variance of item *i*, $\sigma_{T}^{2}$ the total-score variance, *λ_i_* a standardized loading, and *θ_i_* the corresponding error variance: For clarity, CR uses the squared sum of standardized loadings, whereas AVE uses the sum of squared standardized loadings.

(1) $$\alpha \;=\;\frac{k}{k\;-\;1}\;[1\;-\;\frac{\Sigma_{i}{\sigma_{i}}^{2}}{{\sigma_{T}}^{2}}]$$

(2) $$\mathrm{CR}\;=\;\frac{{\left(\Sigma_{i}\lambda_{i}\right)}^{2}}{{\left(\Sigma_{i}\lambda_{i}\right)}^{2}\;+\;\Sigma_{i}\theta_{i}}$$

(3) $$\mathrm{AVE}\;=\;\frac{\Sigma_{i}{\lambda_{i}}^{2}}{\Sigma_{i}{\lambda_{i}}^{2}\;+\;\Sigma_{i}\theta_{i}}$$

A six-factor confirmatory factor analysis (CFA) was then used to test whether the 27 scored indicators represented the six theorized constructs—AIL, PSI, EA, ER, SCJ, and VB—as distinct but correlated factors. Each indicator loaded only on its intended factor, and the latent factors were allowed to correlate. In generic form, the measurement relation was:

*x_i_* = *λ_ij_η_j_* + *ε_i_* (4)

where *x_i_* is an observed indicator, *η_j_* is its intended latent construct, *λ_ij_* is the standardized factor loading, and *ε_i_* is indicator-specific error. Model adequacy was assessed jointly with χ^2^ relative to degrees of freedom, CFI, TLI, RMSEA, and SRMR rather than relying on the χ^2^ statistic alone, which is sensitive to sample size. CR and AVE were considered alongside the standardized loadings, and discriminant validity was examined using factor correlations, the Fornell–Larcker criterion, and HTMT diagnostics. Because epistemic authority and epistemic reliability were theoretically adjacent and displayed the largest latent correlation, an additional nested CFA comparison was conducted as a targeted discriminant-validity check. The retained six-factor measurement model was compared with a more constrained five-factor alternative in which the four EA and four ER indicators were forced to load on a single combined epistemic factor while the remaining constructs retained their original factor assignments. A significant χ^2^ deterioration in the collapsed model was taken as evidence that separating EA and ER captured measurement structure not represented by a single epistemic factor. This sequence established whether the proposed constructs could be treated as empirically distinguishable before their specified association patterns were interpreted.

After establishing measurement adequacy, the proposed relations among the post-interaction constructs were examined using standardized composite-score path models. This stage was used descriptively to quantify the direct associations specified by the theoretical framework while keeping the validated measurement model conceptually separate from the subsequent predictive equations. Except for paths originating in the randomized experimental condition, coefficients among the psychological constructs are associational and are not interpreted as evidence of temporal ordering. All continuous predictors and outcomes were standardized, and the binary experimental condition was standardized from its 0/1 coding. For each endogenous variable *m*, the general equation was:

(5) $${Y_{m}}^{*}\;=\;\beta_{0m}\;+\;\Sigma_{j}\beta_{\mathrm{jm}}{X_{j}}^{*}\;+\;\varepsilon_{m}$$

where $Y_{m}^{*}$ is a standardized endogenous construct, $X_{j}^{*}$ denotes the standardized predictors included in that equation, *β_jm_* is the standardized path coefficient, and *ε_m_* is the residual. Heteroskedasticity-consistent HC3 standard errors were used for path-level inference because composite-score regressions need not have constant residual variance. This provided a more conservative assessment of the hypothesized construct-level associations and reduced dependence on the homoskedasticity assumption. Respondent-level harmful-overreliance and appropriate-reliance rates were used only in exploratory post-interaction association models. They were not used to estimate the primary randomized condition effect on behavior.

Exploratory post-interaction compound associations were evaluated with nonparametric bootstrap estimation. Because the psychological variables were assessed after the behavioral trials, these calculations were used only to determine whether the observed covariance pattern was statistically consistent with the proposed Social Intelligence Reliance Halo framework; they were not treated as indirect causal effects or evidence of mediation of earlier behavioral reliance. Using 5000 bootstrap resamples, the same standardized equations were re-estimated in each resample. For a specified association chain *p* containing *R_p_* component associations, the compound estimate was computed as:

(6) $${\mathrm{CA}}_{p}\;=\;\prod_{r\;=\;1}^{R_{p}}\beta_{r}$$

Percentile 95% bootstrap confidence intervals were used as the primary inferential criterion; a compound association was treated as statistically distinguishable from zero when its interval excluded zero. Component paths were retained even when an individual coefficient was nonsignificant so that the bootstrap distribution, rather than stepwise path deletion, determined the estimated compound association. These analyses were interpreted exploratorily because the post-interaction measures cannot establish that the psychological variables preceded or caused the behavioral reliance observed during the earlier decision trials.

The primary behavioral analysis combined all 2772 decision trials in a population-averaged logistic GEE with an exchangeable within-respondent working-correlation structure and robust sandwich standard errors. The model included randomized relational-framing condition, the participant-dependent correctness of the contingent AI recommendation, their interaction, initial confidence, and trial indicators. The participant-dependent advice-correctness indicator *A_it_* was coded 1 when the participant’s initial answer was wrong and the AI recommendation was therefore correct, and 0 when the initial answer was correct and the AI recommendation was therefore wrong. Importantly, participant-dependent advice correctness was not a second randomized experimental factor: it was mechanically determined by initial-response accuracy because the AI consistently recommended the unselected alternative. Accordingly, the model is not interpreted as a randomized 2 × 2 Condition × AI-Accuracy design, and the interaction is treated as an exploratory treatment-heterogeneity analysis. Post-interaction psychological measures (PSI, EA, ER, SCJ, and VB) were deliberately excluded from this primary treatment-effect model because they were assessed after experimental exposure and after the behavioral decisions. The primary behavioral model was:

logit[P(*S_it_* = 1)] = *γ*_0_ + *γ*_1_*C_i_* + *γ*_2_*A_it_* + *γ*_3_(*C_i_* × *A_it_*) + *γ*_4_Conf*_it_* + *δ*′*T_it_* (7)

where *S_it_* denotes switching, *C_i_* denotes the randomized High-SI condition, *A_it_* denotes participant-dependent advice correctness, Conf*_it_* denotes initial confidence, and *T_it_* represents trial indicators. The condition effect among initially correct trials, where the AI advice was necessarily wrong, provided the primary test of H5; the corresponding condition effect among initially incorrect trials, where the AI advice was necessarily correct, provided the primary test of H6. The interaction coefficient *γ*_3_ addressed ERQ1 by testing whether the randomized High-SI package effect differed across these two participant-dependent correctness states. It does not estimate the interaction of two independently randomized factors. A similar positive condition effect in both states without a reliable interaction would be described as generalized reliance amplification across these participant-dependent states; it would not by itself establish whether reliance responds differently to independently varying AI accuracy. As an exploratory respondent-level summary, the reliance-quality contrast can be expressed as CC*_i_* = AR*_i_* − HOR*_i_*, where AR*_i_* and HOR*_i_* denote respondent *i*’s appropriate-reliance and harmful-overreliance rates, respectively. This index summarizes relative switching across participant-dependent states and is not treated as confirmatory evidence about responsiveness to independently manipulated AI accuracy. Models that additionally included post-interaction psychological variables were retained only as exploratory secondary analyses and are reported in the Supplementary Material; their condition coefficients were not interpreted as primary causal treatment effects.

Finally, robustness analyses examined whether the principal conclusions depended on a particular covariate set, working-correlation assumption, or decision item. Central construct-association paths were re-estimated with HC3 inference after adding age, work experience, managerial status, and decision responsibility, followed by an extended set including education, AI-use frequency, and gender indicators. Trial-level GEE results were compared under exchangeable and independence working-correlation structures, the primary behavioral reliance model was re-estimated with demographic and work controls, and six leave-one-trial-out models assessed whether any single country comparison drove the findings. The respondent-level exploratory reliance-quality contrast was additionally evaluated with 5000 bootstrap resamples and a 5000-permutation test. These sensitivity checks were used to distinguish substantively stable effects from results that might arise from model specification or a particular decision trial.

An exploratory configurational analysis was additionally conducted. The fsQCA membership-calibration anchors and fuzzy-set scores are reported in Table S9, the necessity analysis in Table S10, the truth table and sufficiency screening in Table S11, and the solution with robustness/sensitivity analyses in Table S12 of the Supporting Information.

## 4. Results

The results are reported in an inferential sequence that separates confirmatory from exploratory evidence. Section 4.1 and Section 4.2 establish measurement adequacy and the randomized manipulation check. Section 4.3 reports the post-interaction construct associations for H2a–H4, which are associational rather than causal. Section 4.4 then reports the primary randomized behavioral effects for harmful and appropriate reliance (H5–H6). Section 4.5 contains the exploratory analyses: treatment heterogeneity across participant-dependent correctness states (ERQ1), post-interaction compound associations (ERQ2), and supplementary AI-literacy behavioral and moderation analyses. Section 4.6 evaluates robustness and sensitivity. This ordering is intended to keep the strength of each inference aligned with the design that identifies it.

### 4.1. Preliminary Analysis and Measurement Model

Preliminary analyses were conducted using the final analytic sample of 462 participants. Examination of the focal constructs showed no evidence of problematic univariate distributions. As summarized in Table 2, absolute skewness values did not exceed 0.295 and absolute excess kurtosis did not exceed 0.864. These values were comfortably within the practical screening thresholds adopted for the analysis. The constructs also displayed adequate variability across their respective response ranges. No transformation or other distributional correction was therefore required before measurement-model estimation.

The six-factor confirmatory factor analysis provided good fit to the data, χ^2^(309) = 559.33, χ^2^/df = 1.81, CFI = 0.954, TLI = 0.947, RMSEA = 0.042, and SRMR = 0.046. Standardized factor loadings ranged from 0.561 to 0.872. Composite reliability estimates ranged from 0.797 to 0.891, exceeding the conventional 0.70 criterion for all constructs, while AVE values ranged from 0.502 to 0.591 and therefore met the 0.50 criterion for convergent validity. Taken together, these results supported the reliability and convergent validity of the measurement model.

Discriminant validity was broadly supported, although the analysis identified one marginal Fornell–Larcker exception that is reported transparently. The criterion was satisfied for five of the six constructs. For Epistemic Authority, the square root of AVE (0.709) was marginally lower than its latent correlation with Perceived Epistemic Reliability, *r* = 0.716, a difference of 0.007. The complementary HTMT evidence supported separation: the EA-ER HTMT ratio was 0.715, below the conservative 0.85 threshold, and its bootstrapped 95% confidence interval [0.648, 0.777] remained well below 1.00. All 15 pairwise HTMT ratios were below 0.85, and every bootstrapped upper confidence bound was below 1.00. To examine the EA-ER distinction more directly, the six-factor CFA was compared with a nested five-factor model that collapsed all EA and ER indicators onto one epistemic factor. The collapsed model fit materially worse, χ^2^(314) = 758.28, CFI = 0.918, TLI = 0.908, RMSEA = 0.055, and SRMR = 0.049, compared with χ^2^(309) = 559.33, CFI = 0.954, TLI = 0.947, RMSEA = 0.042, and SRMR = 0.046 for the six-factor model. The nested chi-square difference was substantial, Δχ^2^(5) = 198.95, *p* < .001, providing additional evidence that a single combined EA/ER factor does not reproduce the measurement structure as well as two related factors. Taken together, the content distinction, HTMT results, and nested CFA comparison support retaining epistemic authority and epistemic reliability as separate but closely related constructs, while the marginal Fornell–Larcker exception cautions against portraying them as strongly independent dimensions. More detailed item-level reliability, CFA loading, Fornell–Larcker, and HTMT results are provided in the Supplementary Material. Item-level reliability diagnostics are reported in Table S2 of the Supporting Information; for consistency, its construct-level summary uses the same CFA-based composite reliability estimates reported in Table S3. Detailed CFA loadings, Fornell–Larcker, HTMT, and common-method diagnostics are reported in Table S3.

### 4.2. Manipulation Check

The effectiveness of the experimental manipulation was examined by comparing the Neutral AI condition with the randomized High-SI relational framing package on perceived social intelligence. Because equality of variances was not assumed, Welch’s independent-samples *t*-test was used. The primary manipulation check strongly supported the intended experimental distinction.

Participants assigned to the High-SI relational framing package reported substantially greater perceived social intelligence (M = 3.462, SD = 0.861) than those exposed to the Neutral AI (M = 2.890, SD = 0.936). The mean difference of 0.572 was statistically significant, *t*(456.19) = 6.83, *p* < .001, 95% CI [0.407, 0.736], and corresponded to a moderate effect, Cohen’s d = 0.636. Thus, the experimental framing meaningfully increased perceptions that the AI possessed social intelligence.

Table 3 summarizes the primary and supplementary manipulation-check comparisons across the Neutral condition and the High-SI relational framing package condition.

The supplementary manipulation indicators showed the same pattern. Relative to the Neutral AI condition, participants assigned to the High-SI relational framing package reported feeling more understood, perceived the system as more socially intelligent than a conventional analytical tool, felt more personally understood, and rated the AI as more socially competent. All four supplementary differences were statistically significant at *p* < .001, with Cohen’s d values ranging from 0.446 to 0.581. The convergence between the primary PSI measure and the supplementary indicators provides stronger evidence that the experimental manipulation altered the intended social-cognitive perception, rather than producing an isolated response on a single measure.

Overall, the manipulation check therefore confirmed that the High-SI relational framing package successfully increased perceived social intelligence and related perceptions of interpersonal understanding. This result establishes the experimental manipulation check needed before examining post-interaction epistemic-evaluation associations and the randomized treatment effect on behavioral reliance.

### 4.3. Post-Interaction Construct Associations (H2a–H4)

The proposed construct-level relationships were examined using standardized composite-score path models with HC3 heteroskedasticity-robust standard errors. Apart from the randomized High-SI package effect on perceived social intelligence (H1), the coefficients in this section describe post-interaction associations rather than causal effects. As shown in Table 4, assignment to the High-SI relational framing package significantly increased perceived social intelligence, *β* = 0.302, 95% CI [0.216, 0.388], *p* < .001, supporting H1. Perceived social intelligence was positively associated with both epistemic authority, *β* = 0.394, 95% CI [0.303, 0.485], *p* < .001, and perceived epistemic reliability, *β* = 0.456, 95% CI [0.367, 0.545], *p* < .001. These findings support H2a and H2b as associational hypotheses and show that post-interaction social perceptions covaried with competence-relevant epistemic judgments.

The downstream paths revealed a more differentiated pattern. Epistemic authority was positively associated with suspension of critical judgment, *β* = 0.234, 95% CI [0.132, 0.336], *p* < .001, supporting H3a. In contrast, the corresponding path from perceived epistemic reliability to suspension of critical judgment was positive but not statistically significant, *β* = 0.084, 95% CI [−0.018, 0.186], *p* = .108; H3b was therefore not supported. Perceived social intelligence also showed an additional direct association with suspension of critical judgment, *β* = 0.172, 95% CI [0.073, 0.272], *p* = .001, suggesting that the perceived social quality of the AI retained an association with reduced scrutiny even after the epistemic variables were taken into account. The confidence interval for the ER-to-SCJ coefficient included values from a very small negative association to a modest positive association. Accordingly, the nonsignificant H3b result is interpreted as insufficient evidence for the hypothesized association, not as evidence that epistemic reliability and suspended judgment are equivalent to zero in the population.

AI literacy displayed the expected vigilance-related association pattern. Greater AI literacy was associated with lower suspension of critical judgment, *β* = −0.324, 95% CI [−0.404, −0.244], *p* < .001, and greater verification behavior, *β* = 0.330, 95% CI [0.241, 0.420], *p* < .001. H4 was therefore supported across both indicators of epistemic vigilance. Suspension of critical judgment was itself negatively related to verification behavior, *β* = −0.283, 95% CI [−0.366, −0.201], *p* < .001. Perceived epistemic reliability was likewise associated with reduced verification, *β* = −0.224, 95% CI [−0.303, −0.144], *p* < .001. Thus, greater perceived epistemic reliability was not significantly associated with suspended judgment, but it was associated with a lower tendency to independently verify the system’s information.

The models explained 12.0% of the variance in perceived social intelligence, 14.8% in epistemic authority, 19.8% in epistemic reliability, 22.7% in suspension of critical judgment, and 31.7% in verification behavior. Overall, the observed covariation was consistent with the association pattern proposed by the Social Intelligence Reliance Halo framework, while also showing that epistemic authority and epistemic reliability did not display identical relationships. Epistemic authority was more strongly associated with suspension of judgment, whereas epistemic reliability was more clearly associated with reduced verification and with behavioral reliance observed earlier during the decision trials. These post-interaction relationships do not establish that the psychological variables temporally preceded or caused the behavioral outcomes. Full multicollinearity diagnostics, post-interaction association paths, effect sizes, and predictive-relevance statistics are provided in Table S4 of the Supporting Information.

Table 4 provides an integrated summary of the confirmatory experimental and associational hypotheses together with the exploratory analyses. The detailed interpretations below retain the distinction between randomized effects, post-interaction associations, and exploratory contrasts.

**Figure 2:**
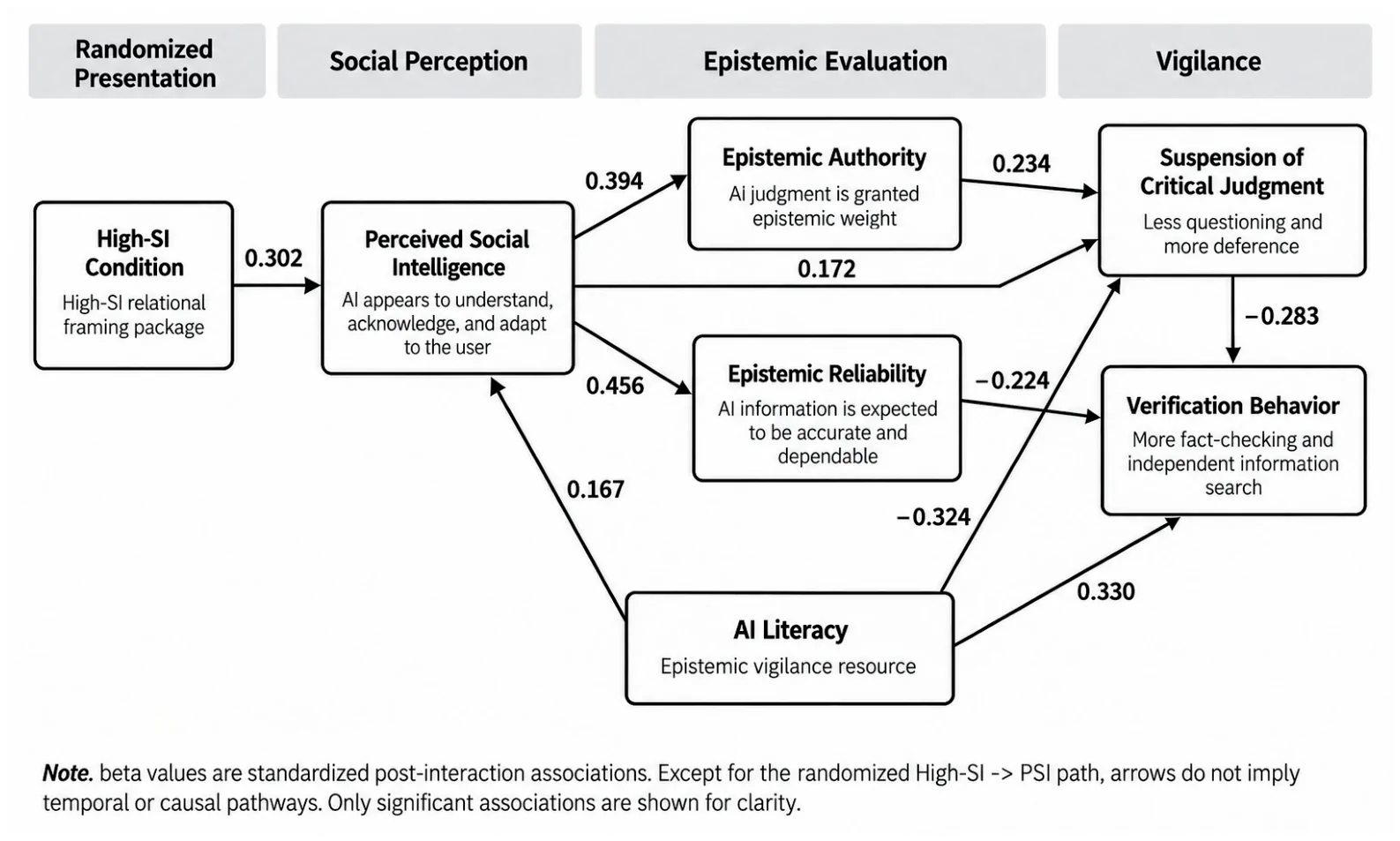
Post-Interaction Construct Associations Relevant to the Social Intelligence Reliance Halo. The High-SI node denotes the randomized relational framing package. Except for the randomized package-to-PSI path, arrows represent standardized post-interaction associations rather than established temporal or causal pathways.

Figure 2 provides a visual summary of the post-interaction construct associations relevant to the Social Intelligence Reliance Halo framework, highlighting how perceived social intelligence was statistically related to epistemic authority, epistemic reliability, critical judgment, and verification behavior. Because these psychological measures were collected after the behavioral trials, arrows among them represent model-specified associations rather than established temporal or causal effects.

### 4.4. Confirmatory Behavioral Reliance Effects (H5–H6)

The behavioral analyses distinguished trials according to participants’ initial accuracy, which mechanically determined whether the AI’s contingent recommendation was harmful or beneficial. Among the 1777 initially correct decisions, the AI recommendation was necessarily wrong because it always endorsed the unselected alternative. Harmful switching occurred on 24.2% of eligible trials in the Neutral condition and 32.3% in the High-SI relational framing package condition. In the primary GEE, the High-SI relational framing package significantly increased the odds of following incorrect AI advice, OR = 1.507, 95% CI [1.200, 1.891], *p* < .001, supporting H5.

Appropriate reliance was evaluated across 995 initially incorrect decisions; on these trials, the unselected alternative recommended by the AI was necessarily correct. Participants switched to the correct AI recommendation on 26.3% of eligible Neutral trials compared with 39.4% of High-SI trials. The same primary GEE showed that the High-SI relational framing package significantly increased the odds of following correct AI advice, OR = 1.800, 95% CI [1.382, 2.346], *p* < .001, supporting H6. Thus, the randomized High-SI relational framing package increased advice-taking in both initially correct and initially incorrect trial subsets, corresponding to harmful and beneficial reliance opportunities, respectively.

Table 5 summarizes the primary randomized behavioral effects for H5 and H6 together with the exploratory participant-dependent correctness-state contrasts used to address ERQ1. Detailed trial-level behavioral models and trial-specific reliance rates are provided in Table S7 of the Supporting Information.

### 4.5. Exploratory Analyses

#### 4.5.1. Exploratory Treatment-Heterogeneity and Reliance-Quality Contrasts (ERQ1)

To examine whether the randomized High-SI package effect varied across participant-dependent correctness states, the Condition × participant-dependent advice-correctness interaction was estimated in the same primary 2772-trial GEE. These correctness states were not experimentally assigned: AI advice was wrong on trials where the participant was initially correct and correct on trials where the participant was initially incorrect. Accordingly, this analysis addresses ERQ1 as an exploratory treatment-heterogeneity comparison. It does not test whether reliance changes as a function of an independently manipulated AI-accuracy factor.

The Condition × participant-dependent advice-correctness interaction was not significant, OR = 1.195, 95% CI [0.860, 1.660], *p* = .289. Thus, there was no reliable evidence that the randomized High-SI package effect differed between initially correct trials (AI wrong) and initially incorrect trials (AI correct). This null interaction should not be interpreted as evidence that High-SI does or does not improve accuracy-sensitive reliance, because recommendation correctness was not independently randomized. The analytic respondent-level reliance-quality contrast between the High-SI and Neutral conditions was likewise small and nonsignificant, difference = 0.039, 95% CI [−0.040, 0.119], *p* = .330, and is treated as an exploratory descriptive comparison. A separate bootstrap/permutation robustness check is reported below and yields the same conclusion. Taken together, these analyses show generalized reliance amplification across the participant-dependent states while leaving the question of responses to independently varied AI accuracy unresolved. The interaction confidence interval (OR = 0.860 to 1.660) spans values compatible with a modestly weaker or stronger High-SI package effect across the two participant-dependent states. Thus, failure to reject the interaction is not an equivalence test and should not be read as evidence that the treatment effects were identical. Likewise, the respondent-level contrast is imprecisely estimated and its confidence interval includes differences in both directions.

This generalized reliance-amplification pattern is the central behavioral evidence relevant to the Social Intelligence Reliance Halo: the High-SI relational framing package increased advice-taking in both participant-defined trial types. The result shows that the framing increased behavioral influence across both beneficial and harmful reliance opportunities in this task. It does not establish whether the High-SI relational framing package makes reliance more or less responsive to independently varied AI accuracy, which would require an orthogonal manipulation of recommendation correctness.

**Figure 3:**
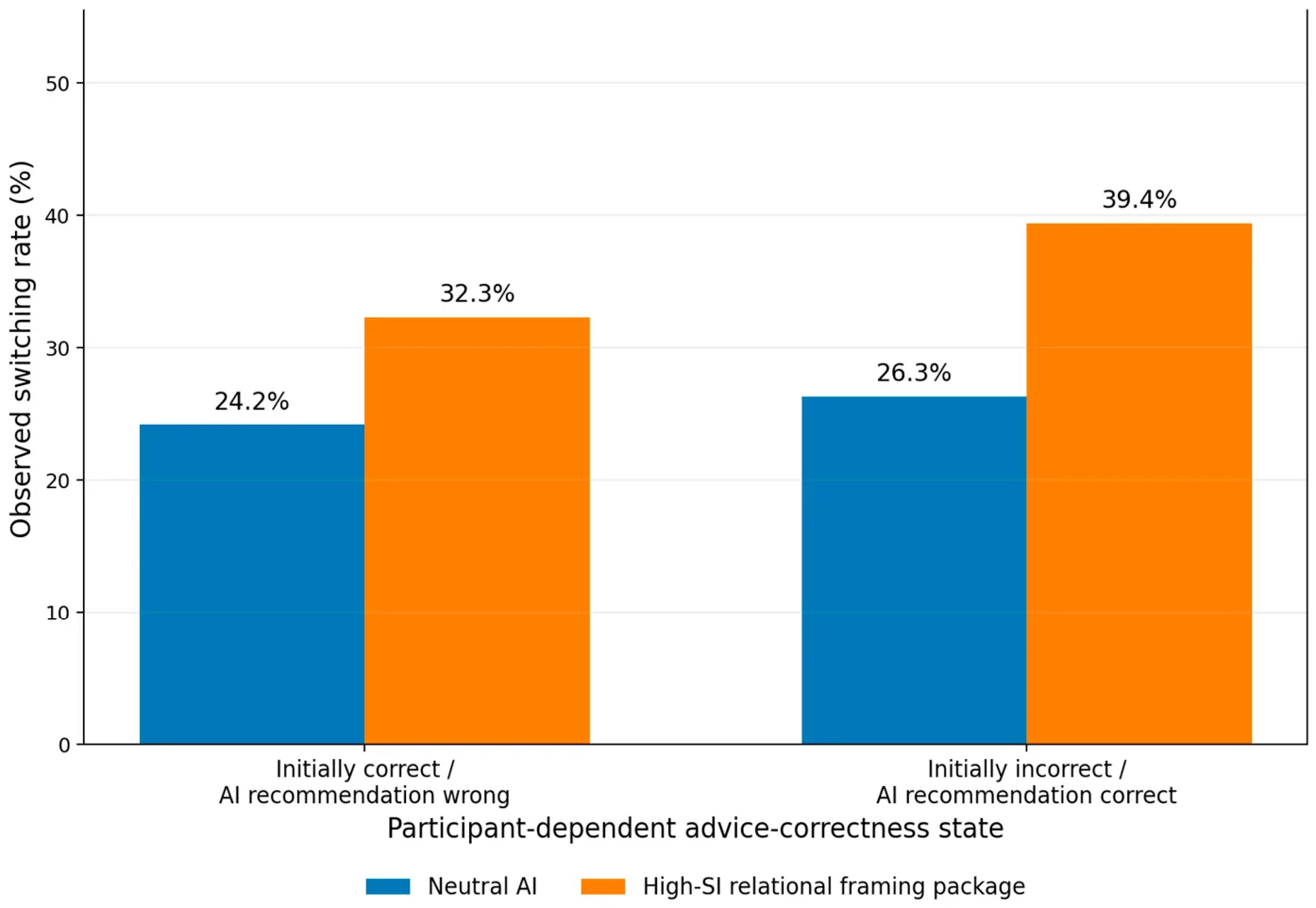
Observed Advice-Taking Rates by Participant-Dependent Advice-Correctness State and Randomized Relational-Framing Condition. Note: Bars show unadjusted observed switching rates: 24.2% (Neutral) and 32.3% (High-SI) when participants were initially correct and the AI recommendation was wrong, and 26.3% (Neutral) and 39.4% (High-SI) when participants were initially incorrect and the AI recommendation was correct. Relational-framing condition was randomized; recommendation correctness was participant-dependent and was not independently randomized. GEE odds ratios are reported in Table 5.

Figure 3 illustrates the exploratory treatment-heterogeneity pattern using the observed, unadjusted switching rates across the two participant-dependent advice-correctness states and the two randomized relational-framing conditions.

#### 4.5.2. Exploratory Post-Interaction Compound Associations (ERQ2)

This section is exploratory and does not test causal mediation. All focal psychological variables were measured after the six behavioral trials; accordingly, the analyses below describe post-interaction compound associations and cannot establish temporal precedence or explain the earlier behavioral effect causally. Using 5000 bootstrap resamples, several statistically nonzero compound associations linked post-interaction perceived social intelligence with harmful overreliance observed during the earlier decision trials. As shown in Table 6, the association chains involving epistemic authority and suspension of critical judgment, suspension of critical judgment alone, and perceived epistemic reliability were statistically distinguishable from zero. The largest specific compound estimate involved perceived epistemic reliability. These estimates are interpreted solely as covariation patterns consistent with the proposed theoretical framework. The complete 5000-resample exploratory compound-association results are reported in Table S5 of the Supporting Information.

The aggregate compound association linking PSI with harmful overreliance was statistically distinguishable from zero, estimate = 0.1266, 95% CI [0.0776, 0.1806], whereas the direct PSI–harmful-overreliance association was nonsignificant. Under ERQ2, this pattern indicates that several post-interaction construct chains covaried with harmful overreliance in the direction anticipated by the theoretical framework. However, the aggregate condition → PSI → downstream compound association was not significant, estimate = 0.0250, 95% CI [−0.0037, 0.0547], *p* = .092. None of these findings establishes that the post-interaction psychological variables mediated, temporally preceded, or caused the randomized experimental effect on the earlier behavioral decisions. The 95% confidence interval for the aggregate condition-to-PSI-to-downstream compound association crossed zero, so the *p* = .092 result does not provide evidence for a statistically distinguishable compound association; it is retained only as an exploratory estimate and not as support for mediation.

#### 4.5.3. Supplementary AI-Literacy Behavioral and Moderation Results

AI literacy showed an association pattern consistent with stronger epistemic vigilance. Higher AI literacy was associated with lower suspension of critical judgment, *β* = −0.324, 95% CI [−0.404, −0.244], *p* < .001, and greater verification behavior, *β* = 0.330, 95% CI [0.241, 0.420], *p* < .001. However, its direct association with harmful behavioral overreliance became nonsignificant once the epistemic and vigilance variables were included in the full exploratory model, *β* = −0.048, *p* = .409. In addition, none of the five moderation interactions involving AI literacy was statistically significant. The results therefore support an association between AI literacy and vigilance-related responses, but not evidence that AI literacy causally produced those responses or systematically buffered the effect of socially intelligent presentation. Because the moderation confidence intervals all included zero, these analyses provide no reliable evidence that AI literacy changed the randomized High-SI package effect in this sample. They should not be interpreted as proving the absence of moderation, particularly because the study was not designed around those interaction effects. Full AI-literacy vigilance and moderation tests are reported in Table S6 of the Supporting Information.

### 4.6. Robustness and Sensitivity Analyses

A series of robustness analyses was conducted to determine whether the principal behavioral findings were sensitive to alternative model specifications, additional covariates, individual decision trials, or alternative inferential procedures. As summarized in Table 7, the results remained highly consistent with the primary analyses. The complete robustness and sensitivity results are provided in Table S8 of the Supporting Information.

First, the primary behavioral reliance model was re-estimated after adding only pre-treatment/background demographic and work-related controls. The High-SI relational framing package continued to increase reliance on initially correct trials, where the contingent AI advice was wrong, OR = 1.488, 95% CI [1.184, 1.869], *p* < .001, and on initially incorrect trials, where the advice was correct, OR = 1.773, 95% CI [1.361, 2.310], *p* < .001. The Condition × participant-dependent advice-correctness interaction remained nonsignificant, OR = 1.192, 95% CI [0.855, 1.661], *p* = .300. The same substantive pattern was obtained under an independence working-correlation structure, with and without these background controls. Models that adjusted for post-interaction PSI, ER, SCJ, or verification were treated separately as exploratory secondary analyses rather than robustness estimates of the randomized treatment effect.

As an additional exclusion-sensitivity analysis, the primary behavioral reliance model was re-estimated using all 482 completed responses, including the 20 respondents who failed the attention check. The substantive pattern was unchanged. The High-SI relational framing package increased advice-taking on initially correct trials, where the contingent AI advice was wrong, OR = 1.491, 95% CI [1.193, 1.865], *p* < .001, and on initially incorrect trials, where the advice was correct, OR = 1.814, 95% CI [1.401, 2.350], *p* < .001. The Condition × participant-dependent advice-correctness interaction remained nonsignificant, OR = 1.217, 95% CI [0.883, 1.676], *p* = .230. Thus, the exclusion of attention-check failures did not account for the main reliance-amplification finding.

Second, six leave-one-trial-out models were estimated. In every specification, the High-SI relational framing package continued to significantly increase reliance on both initially correct trials (AI advice wrong) and initially incorrect trials (AI advice correct), whereas the Condition × participant-dependent advice-correctness interaction remained nonsignificant. Thus, the reliance-amplification pattern was not driven by any single country-comparison task.

Finally, the respondent-level reliance-quality contrast remained nonsignificant under both bootstrap and permutation inference, difference = 0.039, 95% bootstrap CI [−0.038, 0.118], *p* = .340. Taken together, the robustness analyses reinforce the generalized reliance-amplification finding. The exploratory interaction and respondent-level contrast did not detect a reliable differential effect across participant-dependent correctness states, but these results cannot determine whether High-SI framing makes reliance more or less responsive to independently manipulated AI accuracy. These sensitivity checks strengthen the stability of the H5/H6 reliance-amplification results, but they do not convert the exploratory null interaction or reliance-quality contrast into evidence of equivalence or into a valid test of responses to independently randomized AI accuracy.

Table 7 summarizes sensitivity analyses for the primary H5/H6 behavioral effects and the exploratory correctness-state contrasts, allowing the stability of the randomized reliance findings to be evaluated separately from the interpretation of the exploratory reliance-quality analyses.

## 5. Discussion

### 5.1. Randomized Behavioral Effects: Generalized Reliance Amplification

The strongest evidence in the study comes from the randomized contrast between the High Social-Intelligence (High-SI) relational framing package and the Neutral condition. The High-SI package increased advice-taking on initially correct trials, where the contingent AI recommendation was wrong (OR = 1.507, 95% CI [1.200, 1.891], *p* < .001), and on initially incorrect trials, where the contingent AI recommendation was correct (OR = 1.800, 95% CI [1.382, 2.346], *p* < .001). In observed terms, harmful switching increased from 24.2% to 32.3%, while appropriate switching increased from 26.3% to 39.4%. These results support a generalized reliance-amplification interpretation for the randomized package: socially elaborated relational framing increased the behavioral influence of the AI across both participant-dependent correctness states. Because the manipulation was a bundle, this causal statement applies to the package as a whole rather than to any single cue such as perspective acknowledgment, affirmation, personalization, warmth, or message elaboration.

The behavioral pattern also reinforces why total advice acceptance is not an adequate indicator of successful human–AI collaboration. If only initially incorrect trials had been examined, the High-SI condition would appear beneficial because more participants corrected wrong initial judgments by following the AI. Yet the same package also increased switching away from correct initial judgments when the AI’s contingent recommendation was wrong. Greater receptivity can therefore produce both beneficial and harmful behavioral consequences. This distinction is consistent with Lee and See’s (2004) emphasis on appropriate reliance, Parasuraman and Riley’s (1997) distinction between use and misuse, and work showing that algorithm appreciation or aversion is not inherently desirable apart from the quality of the advice being accepted or rejected (Dietvorst et al., 2015; Logg et al., 2019; Mahmud et al., 2022).

### 5.2. The Social Intelligence Reliance Halo and Post-Interaction Evidence

The findings are consistent with the Social Intelligence Reliance Halo as a social-to-epistemic spillover framework, but the evidentiary components should be kept distinct. Experimentally, the randomized High-SI package increased perceived social intelligence and behavioral reliance. Associationally, post-interaction perceived social intelligence was positively related to epistemic authority and epistemic reliability. The theoretical proposition connecting these findings is that a relational impression of being understood can become a non-diagnostic cue for judgments about whether an AI deserves epistemic weight or is likely to be factually dependable. This proposition differs from anthropomorphism, generic trust, algorithm appreciation, advice-taking, and automation bias because it concerns transfer from a social evaluation domain to an epistemic evaluation domain. The present data are consistent with that account, but they do not establish the complete psychological sequence through which the randomized framing package produced the earlier behavioral decisions.

The distinction between epistemic authority and epistemic reliability is important to that framework. Epistemic authority concerns the standing and decisional weight granted to the AI as a knowledge source, whereas epistemic reliability concerns expected factual accuracy, relevance, and dependability. The two constructs were closely related, as theory would predict, but the measurement evidence favored treating them as distinct: the EA-ER HTMT ratio was 0.715 with a bootstrapped upper 95% bound of 0.777, and collapsing their indicators into a single factor significantly worsened model fit, delta chi-square(5) = 198.95, *p* < .001. Their post-interaction association patterns also differed. Epistemic authority was significantly associated with greater suspension of critical judgment, whereas epistemic reliability was not significantly associated with suspension of critical judgment but was associated with lower verification. These findings support conceptual differentiation within the present sample, while remaining associational rather than causal.

The exploratory compound-association analyses provide a further, but deliberately limited, layer of evidence. Several post-interaction construct chains involving perceived social intelligence, epistemic authority or reliability, and suspension of critical judgment were statistically distinguishable from zero in relation to harmful overreliance observed during the earlier trials. However, all focal psychological variables were measured after those behavioral decisions. The analyses therefore cannot establish temporal precedence, causal mediation, or the mechanism responsible for the randomized behavioral effect. The nonsignificant aggregate condition-to-PSI-to-downstream compound association (estimate = 0.0250, 95% CI [−0.0037, 0.0547], *p* = .092) is also not evidence of a mediated treatment effect. The appropriate interpretation is that the post-interaction covariance pattern is compatible with parts of the proposed framework and identifies relationships that should be tested prospectively in designs with temporally ordered measurements.

### 5.3. Participant-Dependent Advice Correctness and Reliance Quality

Reliance quality remains a key normative concern, but the present design does not provide a direct experimental test of whether users adjust reliance to AI accuracy. Because the AI always recommended the unselected alternative, advice correctness was mechanically determined by participants’ initial accuracy: the AI was wrong when the participant was initially correct and correct when the participant was initially incorrect. The Condition × participant-dependent correctness interaction therefore compares the randomized High-SI package effect across two initial-accuracy-defined trial states rather than across independently randomized levels of AI accuracy. The interaction was nonsignificant (OR = 1.195, 95% CI [0.860, 1.660], *p* = .289), but this result does not demonstrate equivalence and does not establish whether High-SI framing makes reliance more or less responsive to independently varying AI accuracy. The confidence interval remains compatible with a modestly weaker or stronger treatment effect across the two states. The respondent-level reliance-quality contrast was likewise exploratory and imprecise. A direct experimental test of accuracy-sensitive reliance requires AI recommendation accuracy to be manipulated independently of participants’ initial knowledge or accuracy.

This distinction changes the interpretation of the central behavioral result without diminishing its importance. The study demonstrates that the High-SI package increased behavioral influence in both beneficial and harmful reliance opportunities. It does not establish whether socially intelligent presentation changes users’ sensitivity to independently varying AI accuracy. Future experiments should therefore cross social-intelligence framing with independently randomized recommendation accuracy so that changes in advice-taking can be attributed separately to relational presentation, objective advice quality, and their interaction. Such a design would permit a direct test of whether social cues increase generalized receptivity or improve discrimination between advice that should and should not be followed.

### 5.4. AI Literacy and Organizational Implications

AI literacy showed a post-interaction pattern consistent with stronger epistemic vigilance. Higher evaluative AI literacy was associated with lower suspension of critical judgment and greater verification behavior, but it did not significantly moderate the randomized High-SI package effect in the supplementary analyses. These findings therefore support an association between literacy and vigilance-related responses, not a causal claim that literacy produced those responses or protected participants from socially persuasive framing. This distinction is consistent with contemporary conceptions of AI literacy that emphasize critical evaluation of capabilities, limitations, and outputs rather than tool use alone (Laupichler et al., 2022; D. T. K. Ng et al., 2021; Wang et al., 2023).

For organizational practice, the most defensible implication concerns the influence of the relational package actually tested. The experiment shows that a package combining perspective acknowledgment, affirmation, personalization, relational warmth, and greater elaboration can increase advice-taking even when it adds no diagnostic factual information. It does not show that any one of those elements independently causes overreliance. Organizations should therefore be cautious about treating socially responsive interface design as epistemic evidence and may benefit from separating relational quality from explicit indicators of factual confidence, source quality, uncertainty, and evidentiary support. In higher-stakes settings, verification prompts, source visibility, independent-check procedures, or modest decision friction may help preserve scrutiny, but these interventions should themselves be evaluated experimentally rather than assumed to offset the influence observed here.

### 5.5. Limitations and Future Research

Several limitations define the boundary of the present conclusions. First, the six repeated tasks involved objectively verifiable population judgments. This feature made beneficial and harmful switching observable, but it does not represent the uncertainty, ambiguity, incentives, accountability, or domain knowledge involved in many organizational decisions. Replication is therefore needed in settings such as finance, hiring, healthcare, procurement, risk management, and strategic decision making. Second, recommendation correctness was participant-dependent rather than independently randomized. Consequently, the exploratory Condition × correctness interaction and respondent-level reliance-quality contrast cannot determine whether the High-SI package makes reliance more or less responsive to independently varying AI accuracy. Third, the High-SI manipulation was a composite package. Perspective acknowledgment, affirmation, personalization, relational warmth or tone, and message elaboration or length changed together, so the experiment identifies the causal effect of the complete package relative to the Neutral comparator, not the unique contribution of social understanding or any individual component.

A fourth limitation concerns temporal ordering. PSI, epistemic authority, epistemic reliability, suspension of critical judgment, and verification behavior were measured after completion of the behavioral trials. Their relationships with earlier reliance are therefore post-interaction associations and cannot establish mediation or direction of influence. Designs intended to test the proposed psychological sequence should measure candidate process variables before or between behavioral trials or experimentally manipulate the relevant epistemic judgments or vigilance processes. A fifth limitation concerns measurement. The EA and ER instruments were developed for the present generative-AI context, although they were grounded in prior theory, reviewed by experts, piloted, and supported by CFA, HTMT, and the nested factor comparison. The present evidence supports their use and separation in this sample, but independent scale-development work should examine their nomological, criterion, convergent, and discriminant validity across populations, decision domains, and AI systems.

Future research can address these limitations with designs that independently manipulate relational framing and AI accuracy, match message length and informational detail across conditions, and isolate specific social cues through factorial or single-cue manipulations. Perspective acknowledgment, affirmation or positive feedback, personalization, relational warmth, and elaboration can be crossed or varied separately to estimate their unique and interactive effects. Longitudinal and repeated-interaction designs can test whether relational influence persists after visible AI errors or whether users adjust reliance with experience. Studies that temporally separate social-perception, epistemic-evaluation, vigilance, and behavioral measures would also provide a stronger basis for testing the proposed process. Extensions to different levels of domain expertise, uncertainty disclosure, confidence signaling, source visibility, and real organizational decisions would clarify when relationally intelligent presentation supports productive collaboration and when it creates epistemic risk. Designs that independently vary recommendation accuracy, as in McGrath et al. (2024), provide a stronger basis for testing accuracy-sensitive reliance because advice quality is not mechanically tied to the participant’s initial performance.

### 5.6. Theoretical Contribution

Within these boundaries, the study contributes a more precise way of conceptualizing socially persuasive AI. The Social Intelligence Reliance Halo does not posit that social intelligence, trust, advice-taking, and overreliance are the same construct. Instead, it proposes that social understanding can function as a non-diagnostic cue that spills over into epistemic evaluation. The randomized findings establish that the relational framing package increased behavioral influence, while the post-interaction findings show that perceived social intelligence covaried with differentiated judgments of epistemic authority and reliability. By separating appropriate reliance from harmful overreliance, the study also demonstrates why greater persuasiveness cannot be equated with better human–AI decision quality. What remains unresolved is whether socially intelligent presentation changes users’ responsiveness to independently varying AI accuracy and whether the proposed post-interaction associations form the causal sequence implied by the theory. Those questions define the next empirical tests of the framework rather than conclusions established by the present design.

## 6. Conclusions

This study examined whether a socially intelligent relational presentation changes the quality of human–AI reliance or primarily increases the behavioral influence of AI advice. The randomized High-SI relational framing package increased advice-taking both when participants were initially wrong and the AI’s alternative was correct and when participants were initially correct and the AI’s alternative was wrong. The strongest experimental conclusion is therefore one of generalized reliance amplification within the present design. The nonsignificant Condition × participant-dependent correctness interaction provides no reliable evidence that the treatment effect differed across these two trial types; however, because recommendation correctness was mechanically determined by initial participant accuracy rather than independently randomized, this result cannot establish whether socially intelligent presentation makes reliance more or less responsive to independently varying AI accuracy.

The theoretical contribution is to specify the Social Intelligence Reliance Halo as a social-to-epistemic spillover framework rather than as another label for anthropomorphism, general trust, or advice acceptance. The framework proposes that perceived social understanding can function as a non-diagnostic cue for epistemic authority and epistemic reliability. Consistent with that account, post-interaction perceived social intelligence was positively associated with both epistemic judgments, and the two constructs showed differentiated relationships with vigilance-related outcomes. Yet these measures were collected after the behavioral trials. Their associations therefore show covariation consistent with the framework, not temporal precedence, causal mediation, or the psychological mechanism that produced the randomized behavioral effect. Keeping these forms of evidence separate is central to the interpretation of the study.

The findings also reinforce the importance of distinguishing reliance quantity from reliance quality. Following AI advice can be beneficial when the recommendation corrects an initial error and harmful when it overturns an initially correct judgment. For this reason, adoption, trust, or overall advice acceptance should not be treated as sufficient indicators of successful human–AI collaboration. A key normative criterion is whether reliance tracks independent differences in recommendation accuracy, but the present experiment only motivates that question; a direct test requires AI accuracy to be manipulated independently of participants’ prior knowledge or initial performance. AI literacy likewise showed potentially relevant post-interaction associations—lower suspension of critical judgment and greater verification—but these findings should be interpreted as correlational evidence about epistemic vigilance rather than proof of a protective causal effect.

The practical implication is that socially responsive AI interfaces should be evaluated not only for engagement and usability but also for the quality of the reliance they induce. Organizations deploying generative AI in managerial, analytical, or operational decisions may benefit from design features that make evidentiary support, uncertainty, provenance, and opportunities for verification visible rather than relying on relational fluency as a proxy for competence. Human oversight remains especially important when an interface can make advice more persuasive without supplying additional diagnostic information about factual correctness.

The treatment itself also defines an important boundary on interpretation. Perspective acknowledgment, affirmation, personalization, relational warmth or tone, and message elaboration or length covaried in the High-SI package, so the experiment identifies the effect of that complete package relative to the Neutral comparator and cannot isolate the contribution of any single component. Future studies should match message length and informational content, manipulate specific relational cues factorially or individually, separate candidate process measures temporally from behavioral outcomes, and independently randomize AI accuracy. Such designs can determine whether a more tightly isolated social-intelligence cue truly helps users recognize when AI deserves epistemic weight. The central challenge is not to maximize reliance, but to preserve the benefits of socially responsive interaction without allowing relational appeal to substitute for epistemic justification.

## Figures and Tables

**Table 1:** Experimental Materials, Survey Measures, and Operational Definitions.

Code/Condition	Construct/Task	Item Wording/Stimulus	Response/Scoring	Source/Role
Panel A. Experimental decision tasks				
D1	Population judgment	Which country had the larger total population in 2025? Germany or Colombia?	Country choice + confidence 0–100	Correct answer: Germany
D2	Population judgment	Which country had the larger total population in 2025? Spain or Poland?	Country choice + confidence 0–100	Correct answer: Spain
D3	Population judgment	Which country had the larger total population in 2025? Argentina or Canada?	Country choice + confidence 0–100	Correct answer: Argentina
D4	Population judgment	Which country had the larger total population in 2025? Malaysia or Australia?	Country choice + confidence 0–100	Correct answer: Malaysia
D5	Population judgment	Which country had the larger total population in 2025? Mexico or Japan?	Country choice + confidence 0–100	Correct answer: Mexico
D6	Population judgment	Which country had the larger total population in 2025? France or Italy?	Country choice + confidence 0–100	Correct answer: France
Panel B. Experimental manipulation scripts				
STIM_HIGH	High-SI relational framing package	“I can see why you may have chosen [INITIAL CHOICE], and your initial judgment is understandable. Taking your perspective into account while focusing specifically on total population, I would recommend [OTHER COUNTRY] as the larger market.”	Displayed after each initial decision	Study-specific; relational social-intelligence cue bundle (perspective acknowledgment, affirmation, personalization)
STIM_NEUTRAL	Neutral AI	“Your initial choice has been recorded. The task requires comparing the two markets using total population. Based on that criterion, I would recommend [OTHER COUNTRY] as the larger market.”	Displayed after each initial decision	Study-specific; neutral analytic framing
Panel C. Core survey measures				
AIL1–AIL3	Evaluative AI Literacy (AIL)	AIL1. After using an AI application for some time, I can evaluate its important capabilities and limitations. AIL2. When an AI system provides several possible solutions, I can evaluate which solution is most appropriate. AIL3. I can determine which type of AI application is appropriate for a particular task.	1 = Strongly disagree to 7 = Strongly agree; mean score	Adapted from Wang et al. (2023)
PSI1–PSI8	Perceived Social Intelligence (PSI)	PSI1. The AI assistant seemed socially aware. PSI2. The AI assistant seemed able to understand why I might make a particular choice. PSI3. The AI assistant seemed able to understand my perspective. PSI4. The AI assistant appeared to adapt its responses to my decisions. PSI5. The AI assistant seemed able to infer what I might be thinking about the alternatives. PSI6. The AI assistant seemed able to anticipate how I might react to its recommendation. PSI7. The AI assistant showed awareness of my reasoning when responding to me. PSI8. The AI assistant seemed socially unaware of the person making the decision.	1 = Strongly disagree to 5 = Strongly agree; PSI8 reverse-coded; mean score	Adapted from Barchard et al. (2020)
EA1–EA4	Epistemic Authority Attribution (EA)	EA1. I would regard this AI assistant as an authoritative source when making this type of decision. EA2. The AI assistant’s opinion would carry substantial weight for me in a professional discussion. EA3. I would tend to treat conclusions from this AI assistant as objective evidence. EA4. For this type of problem, I would rarely feel a strong need to challenge the AI assistant’s authority.	1 = Strongly disagree to 6 = Strongly agree; mean score	Study-developed for the GenAI context; informed by Raviv et al. (1993)
ER1–ER4	Perceived Epistemic Reliability (ER)	ER1. I would expect this AI assistant to provide factually accurate recommendations. ER2. I would expect information from this AI assistant to be relevant and reliable. ER3. I could use this AI assistant as a dependable reference point when seeking information. ER4. When this AI assistant communicates with technical precision, I am inclined to regard its information as accurate.	1 = Strongly disagree to 6 = Strongly agree; mean score	Study-developed for the GenAI context; informed by Jian et al. (2000)
SCJ1–SCJ4	Suspension of Critical Judgment (SCJ)	SCJ1. I could use a recommendation from this AI assistant without independently checking it first. SCJ2. When this AI assistant sounds confident, I feel less need to critically evaluate its recommendation. SCJ3. I would be unlikely to question the AI assistant’s conclusion unless something seemed obviously wrong. SCJ4. I could act on information from this AI assistant without consulting another source.	1 = Strongly disagree to 6 = Strongly agree; mean score; higher = more suspended scrutiny	Study-developed for the GenAI context; informed by Sperber et al. (2010) and Merritt et al. (2019)
VB1–VB4	Verification Behavior (VB)	VB1. I cross-check important AI-generated information against an independent source. VB2. I verify important factual claims made by AI using authoritative or primary sources. VB3. For an important decision, I seek confirmation from another knowledgeable person when necessary. VB4. Before using AI-generated evidence in my work, I check the underlying source, citation, or data when one is provided.	1 = Never to 5 = Always; mean score	Study-developed for the GenAI context; informed by Flanagin and Metzger (2000) and Metzger et al. (2003)
Panel D. Supplementary post-task and quality-control items				
GR1–GR6	General reactions/manipulation checks	GR1. I would be willing to use this AI assistant for similar decision-making tasks in the future. GR2. The AI assistant made me feel that it understood the person making the decision. GR3. The AI assistant appeared more socially intelligent than a conventional analytical tool. GR4. I felt personally understood by the AI assistant. GR5. I regarded the AI assistant as technically competent. GR6. I regarded the AI assistant as socially competent.	1 = Strongly disagree to 7 = Strongly agree	Study-specific secondary outcomes/manipulation checks
AC1	Attention check	This item is included to confirm that you are reading the questions carefully. Please select Agree.	Strongly disagree to Strongly agree; fail if response ≠ Agree	Study-specific quality-control item

**Table 2:** Descriptive Statistics and Measurement-Model Assessment.

Panel A. Construct Descriptives and Convergent Validity						
Construct	M	SD	Skewness	Excess Kurtosis	CR	AVE
Evaluative AI Literacy (AIL)	4.232	1.484	−0.095	−0.743	0.812	0.591
Perceived Social Intelligence (PSI)	3.177	0.943	−0.248	−0.728	0.891	0.508
Epistemic Authority (EA)	3.788	1.202	−0.166	−0.801	0.797	0.502
Perceived Epistemic Reliability (ER)	3.814	1.225	−0.295	−0.575	0.828	0.552
Suspension of Critical Judgment (SCJ)	3.758	1.207	−0.044	−0.864	0.803	0.507
Verification Behavior (VB)	3.052	0.976	0.015	−0.694	0.820	0.534
Panel B. Confirmatory Factor Analysis Model Fit						
Fit Index	Observed Value			Evaluation Criterion	Assessment	
χ^2^	559.33			—	Reported with df	
df	309			—	—	
χ^2^/df	1.81			<3.00	Good	
CFI	0.954			≥0.90; ≈0.95 strong	Good	
TLI	0.947			≥0.90	Good	
RMSEA	0.042			≤0.06 strong	Good	
SRMR	0.046			≤0.08	Good	
Standardized loadings	0.561–0.872			Preferably ≥ 0.50	Acceptable	
Composite reliability	0.797–0.891			≥0.70	Supported	
AVE	0.502–0.591			≥0.50	Supported	
EA+ER collapsed model	χ^2^(314) = 758.28			Alternative five-factor CFA	Worse fit	
Nested CFA comparison	Δχ^2^(5) = 198.95, *p* < .001			Six-factor vs. EA+ER collapsed	Supports separation	

Note. *N* = 462. CR = composite reliability; AVE = average variance extracted; CFI = comparative fit index; TLI = Tucker–Lewis index; RMSEA = root mean square error of approximation; SRMR = standardized root mean square residual; — = not applicable.

**Table 3:** Experimental Manipulation-Check Results.

Measure	Neutral AI *M* (SD)	High-SI Package *M* (SD)	Mean Difference	Welch *t*	*p*	95% CI	Cohen’s *d*
PSI composite	2.890 (0.936)	3.462 (0.861)	0.572	6.83	<.001	[0.407, 0.736]	0.636
Felt understood (GR2)	4.057 (2.002)	5.065 (1.826)	1.008	5.65	<.001	[0.658, 1.359]	0.526
More socially intelligent than conventional tool (GR3)	4.187 (1.979)	5.103 (1.728)	0.916	5.30	<.001	[0.577, 1.256]	0.494
Personally understood (GR4)	4.022 (1.842)	4.845 (1.852)	0.823	4.79	<.001	[0.485, 1.161]	0.446
Socially competent (GR6)	4.026 (1.935)	5.099 (1.754)	1.073	6.24	<.001	[0.735, 1.411]	0.581

Note. Neutral AI: *n* = 230; High-SI relational framing package: *n* = 232. Mean differences are calculated as High-SI minus Neutral. PSI was measured on a 1–5 scale; supplementary manipulation-check items were measured on 1–7 scales. Welch’s independent-samples *t*-tests were used throughout.

**Table 4:** Summary of Confirmatory Hypotheses and Exploratory Analyses.

Hypothesis	Relationship/Test	Estimate	95% CI	*p*	Decision
Panel A—Confirmatory randomized and post-interaction association hypotheses					
H1	Randomized High-SI package → PSI	*β* = 0.302	[0.216, 0.388]	<.001	Supported
H2a	PSI → EA	*β* = 0.394	[0.303, 0.485]	<.001	Supported
H2b	PSI → ER	*β* = 0.456	[0.367, 0.545]	<.001	Supported
H3a	EA → SCJ	*β* = 0.234	[0.132, 0.336]	<.001	Supported
H3b	ER → SCJ	*β* = 0.084	[−0.018, 0.186]	.108	Not supported
H4	AIL → SCJ	*β* = −0.324	[−0.404, −0.244]	<.001	Supported
H4	AIL → VB	*β* = 0.330	[0.241, 0.420]	<.001	Supported
Additional association	PSI → SCJ	*β* = 0.172	[0.073, 0.272]	.001	Significant
Additional association	SCJ → VB	*β* = −0.283	[−0.366, −0.201]	<.001	Significant
Additional association	ER → VB	*β* = −0.224	[−0.303, −0.144]	<.001	Significant
Panel B—Exploratory post-interaction compound associations (ERQ2)					
ERQ2 (expl.)	PSI → EA → SCJ → OR	0.0129	[0.0025, 0.0276]	.012	Statistically nonzero exploratory association
ERQ2 (expl.)	PSI → SCJ → OR	0.0239	[0.0039, 0.0519]	.012	Statistically nonzero exploratory association
ERQ2 (expl.)	PSI → ER → OR	0.0755	[0.0325, 0.1240]	<.001	Statistically nonzero exploratory association
ERQ2 (expl.)	PSI → all specified post-interaction variables → OR	0.1266	[0.0776, 0.1806]	<.001	Statistically nonzero exploratory association
Context	Direct PSI → OR	*β* = −0.0439	[−0.1440, 0.0557]	.393	Not significant
Supplementary	Condition → PSI → all downstream post-interaction paths → OR	0.0250	[−0.0037, 0.0547]	.092	Not significant
Panel C—Confirmatory behavioral effects (H5–H6) and exploratory correctness-state contrasts (ERQ1)					
H5	High-SI package effect: initially correct trials (AI recommendation wrong)	OR = 1.507	[1.200, 1.891]	<.001	Supported
H6	High-SI package effect: initially incorrect trials (AI recommendation correct)	OR = 1.800	[1.382, 2.346]	<.001	Supported
ERQ1	Condition × participant-dependent advice correctness (exploratory treatment-heterogeneity test)	OR = 1.195	[0.860, 1.660]	.289	No reliable differential effect
Exploratory	Respondent-level reliance-quality contrast: High-SI−Neutral	0.039	[−0.040, 0.119]	.330	No reliable difference; exploratory

Note. PSI = perceived social intelligence; EA = epistemic authority; ER = perceived epistemic reliability; SCJ = suspension of critical judgment; VB = verification behavior; AIL = AI literacy; OR in Panel B = harmful overreliance; OR in Panel C = odds ratio. Panel A coefficients are standardized estimates; except for the randomized High-SI package effect on PSI (H1), they describe post-interaction associations rather than causal effects. Panel B reports exploratory post-interaction bootstrap compound associations based on 5000 resamples and should not be interpreted as indirect causal effects or temporal mediation. Panel C reports primary H5/H6 trial-level GEE estimates together with the exploratory Condition × participant-dependent correctness interaction and respondent-level reliance-quality contrast. Detailed secondary models and diagnostic statistics are reported in the Supplementary Material. Recommendation correctness in Panel C was participant-dependent rather than randomized: advice was wrong on initially correct trials and correct on initially incorrect trials. Therefore, the ERQ1 interaction and reliance-quality contrast do not constitute tests of responsiveness to independently manipulated AI accuracy.

**Table 5:** Primary Behavioral Reliance Effects and Exploratory Participant-Dependent Correctness-State Contrasts.

Test	Sample/Comparison	Estimate	95% CI	*p*	Interpretation
Panel A—Primary trial-level behavioral effects					
Harmful reliance: Neutral	1777 initially correct trials	24.2%	—	—	Observed switching rate
Harmful reliance: High-SI	1777 initially correct trials	32.3%	—	—	Observed switching rate
H5: High-SI package effect on initially correct trials (participant-dependent state: AI recommendation wrong)	Primary GEE	OR = 1.507	[1.200, 1.891]	<.001	H5 supported
Appropriate reliance: Neutral	995 initially incorrect trials	26.3%	—	—	Observed switching rate
Appropriate reliance: High-SI	995 initially incorrect trials	39.4%	—	—	Observed switching rate
H6: High-SI package effect on initially incorrect trials (participant-dependent state: AI recommendation correct)	Primary GEE	OR = 1.800	[1.382, 2.346]	<.001	H6 supported
Panel B—Exploratory treatment-heterogeneity and reliance-quality contrasts					
Condition × participant-dependent advice-correctness state	2772 trials	OR = 1.195	[0.860, 1.660]	.289	No reliable differential High-SI package effect (ERQ1)
Exploratory reliance-quality contrast: High-SI−Neutral	Respondent-level contrast	0.039	[−0.040, 0.119]	.330	No reliable difference; exploratory

Note. OR = odds ratio. Observed percentages are unadjusted condition-specific switching rates; odds ratios are derived from the primary trial-level GEE. Recommendation correctness was participant-dependent rather than independently randomized: because the AI always recommended the unselected alternative, advice was wrong on initially correct trials and correct on initially incorrect trials. Panel B is exploratory and should not be interpreted as a test of responsiveness to independently varied AI accuracy.

**Table 6:** Exploratory Post-Interaction Compound Associations with Harmful Overreliance.

Exploratory Association Pattern	Bootstrap Compound Estimate	95% Bootstrap CI	*p*	Interpretation
PSI → EA → SCJ → OR	0.0129	[0.0025, 0.0276]	.012	Statistically nonzero exploratory association
PSI → SCJ → OR	0.0239	[0.0039, 0.0519]	.012	Statistically nonzero exploratory association
PSI → ER → OR	0.0755	[0.0325, 0.1240]	<.001	Statistically nonzero exploratory association
Aggregate PSI compound association	0.1266	[0.0776, 0.1806]	<.001	Statistically nonzero exploratory association
Direct PSI–OR association	−0.0439	[−0.1440, 0.0557]	.393	Not statistically significant
Condition → PSI → downstream post-interaction associations → OR	0.0250	[−0.0037, 0.0547]	.092	Not statistically significant

Note. PSI = perceived social intelligence; EA = epistemic authority; ER = perceived epistemic reliability; SCJ = suspension of critical judgment; OR = harmful overreliance. Compound estimates are based on 5000 bootstrap resamples. Because PSI, EA, ER, and SCJ were measured after the behavioral trials, these estimates describe post-interaction statistical associations only; they do not establish temporal precedence, indirect causal effects, or mediation of the earlier behavioral outcomes.

**Table 7:** Robustness Analyses of Behavioral Reliance and Exploratory Participant-Dependent Correctness-State Contrasts.

Robustness Specification	Initially Correct/AI Wrong High-SI Effect	Initially Incorrect/AI Correct High-SI Effect	Condition × Correctness-State Interaction	Reliance-Quality Contrast	Interpretation
Primary exchangeable GEE	OR = 1.507 95% CI [1.200, 1.891] *p* < .001	OR = 1.800 95% CI [1.382, 2.346] *p* < .001	OR = 1.195 95% CI [0.860, 1.660] *p* = .289	—	Primary pattern supported
All 482 completers (including 20 attention-check failures)	OR = 1.491 95% CI [1.193, 1.865] *p* < .001	OR = 1.814 95% CI [1.401, 2.350] *p* < .001	OR = 1.217 95% CI [0.883, 1.676] *p* = .230	—	Pattern unchanged; exclusions not driving results
Primary exchangeable GEE + demographic/work controls	OR = 1.488 95% CI [1.184, 1.869] *p* < .001	OR = 1.773 95% CI [1.361, 2.310] *p* < .001	OR = 1.192 95% CI [0.855, 1.661] *p* = .300	—	Pattern unchanged
Independence correlation	OR = 1.499 95% CI [1.195, 1.881] *p* < .001	OR = 1.818 95% CI [1.396, 2.369] *p* < .001	OR = 1.213 95% CI [0.873, 1.684] *p* = .249	—	Pattern unchanged
Controls + independence	OR = 1.481 95% CI [1.179, 1.861] *p* < .001	OR = 1.788 95% CI [1.373, 2.329] *p* < .001	OR = 1.207 95% CI [0.867, 1.681] *p* = .266	—	Pattern unchanged
Leave-one-trial-out, D1–D6	OR range = 1.380–1.589 all *p* ≤ .011	OR range = 1.640–1.896 all *p* ≤ .002	OR range = 1.086–1.361 all *p* > .10	—	Same result in all six models
Bootstrap/permutation reliance-quality contrast	—	—	—	High-SI − Neutral = 0.039 95% CI [−0.038, 0.118] *p* = .340	No reliable respondent-level difference; exploratory

Note. OR = odds ratio. GEE = generalized estimating equation. The all-completer sensitivity analysis reintroduced the 20 respondents excluded for failing the instructed-response attention check (*N* = 482). The leave-one-trial-out row summarizes six models, each excluding one of the six decision trials. The respondent-level reliance-quality contrast used 5000 bootstrap resamples and a 5000-permutation test. Because recommendation correctness was participant-dependent rather than independently randomized, the interaction and reliance-quality contrast are exploratory and do not constitute tests of responsiveness to independently varied AI accuracy. Full coefficients and model diagnostics are reported in the Supplementary Material.

## Data Availability

The original contributions presented in this study are included in the article/Supplementary Material. Further inquiries can be directed to the corresponding author.

## Supplementary Materials

The following supporting information can be downloaded at: https://www.mdpi.com/article/10.3390/jintelligence14100248/s1, The following supporting information accompanies this article. The workbook contains twelve worksheets named Table S1 through Table S12, matching the table numbers and titles listed below. Each table is cited in the corresponding Methods or Results section of the manuscript. Table S1. Questionnaire Items, Response Scales, and Scoring. Table S2. Item-Level Reliability Diagnostics. Table S3. CFA, Fornell–Larcker, HTMT, and Common-Method Diagnostics. Table S4. Multicollinearity, Full Post-Interaction Association Paths, Effect Sizes, and Predictive Relevance. Table S5. Exploratory Post-Interaction Compound Association Results. Table S6. AI-Literacy Vigilance and Moderation Tests. Table S7. Trial-Level Behavioral Models and Trial-Specific Reliance Rates. Table S8. Robustness and Sensitivity Analyses. Table S9. fsQCA Membership Calibration Anchors and Fuzzy-Set Scores. Table S10. fsQCA Necessity Analysis. Table S11. fsQCA Truth Table and Sufficiency Screening. Table S12. fsQCA Solution and Robustness/Sensitivity.
